# The TaCl_5_-Mediated Reaction of Dimethyl 2-Phenylcyclopropane-1,1-dicarboxylate with Aromatic Aldehydes as a Route to Substituted Tetrahydronaphthalenes

**DOI:** 10.3390/molecules29122715

**Published:** 2024-06-07

**Authors:** Tat’yana P. Zosim, Rita N. Kadikova, Roman A. Novikov, Alexander A. Korlyukov, Oleg S. Mozgovoj, Ilfir R. Ramazanov

**Affiliations:** 1 Institute of Petrochemistry and Catalysis of Russian Academy of Sciences, Prospekt Oktyabrya 141, 450075 Ufa, Russia; tania-ygnty@ya.ru (T.P.Z.); skill15@mail.ru (O.S.M.); 2 N.D. Zelinsky Institute of Organic Chemistry of Russian Academy of Sciences, Leninsky prospect 47, 119991 Moscow, Russia; roman@ioc.ac.ru; 3 A.N. Nesmeyanov Institute of Organoelement Compounds of Russian Academy of Sciences, Vavilova St., 28 bld. 1, 119334 Moscow, Russia; alex@xrlab.ineos.ac.ru

**Keywords:** aromatic aldehydes, carbocycles, donor–acceptor cyclopropanes, tantalum (V) trichloride, tetrahydronaphthalenes

## Abstract

It is found that the reaction of dimethyl 2-phenylcyclopropane-1,1-dicarboxylate with 2 equivalents each of aromatic aldehydes and TaCl_5_ in 1,2-dichloroethane at 23 °C for 24 h after hydrolysis gives substituted 4-phenyl-3,4-dihydronaphtalene-2,2(1*H*)-dicarboxylates in good yield. This represents a new type of reactions between 2-arylcyclopropane-1,1-dicarboxylates and aromatic aldehydes, yielding chlorinated tetrahydronaphthalenes with a *cis* arrangement of the aryl and chlorine substituents in the cyclohexene moiety. A plausible reaction mechanism is proposed.

## 1. Introduction

In the last 10–15 years, interest in the chemistry of donor–acceptor cyclopropanes (DACs) has grown sharply [1,2,3,4,5]. Their ring-opening transformations are widely used in organic synthesis to assemble various carbo- and heterocyclic compounds, including natural compounds and their analogs [6,7,8,9,10]. Currently, there are a variety of different types of DAC reactions being reported [11]. The (3 + 2) cycloaddition of DACs with aldehydes is a promising synthetic strategy that has been widely used to produce polysubstituted tetrahydrofurans [12,13,14,15,16,17,18]. Diverse aryl-, hetaryl-, alkenyl- and alkyl-substituted DACs were used. The resulting substituted tetrahydrofurans formed with high cis-diastereoselectivity. A broad range of Lewis acids have been investigated using the reaction of 2-phenylcyclopropane-1,1-dicarboxylate with benzaldehyde. Triflates of tin(II), hafnium(IV), copper(II), cerium(II), ytterbium, scandium and zinc showed the greatest activity in the reaction. Tin(IV), titanium(IV) and aluminum chlorides turned out to be useful for involving less active vinyl- and alkyl-substituted DACs and aliphatic aldehydes in the reaction [19]. However, the use of GaCl_3_ as a Lewis acid caused the reaction to take a different route, resulting in the formation of substituted indenes and polycyclic lactones. [20].

**Scheme 1:**
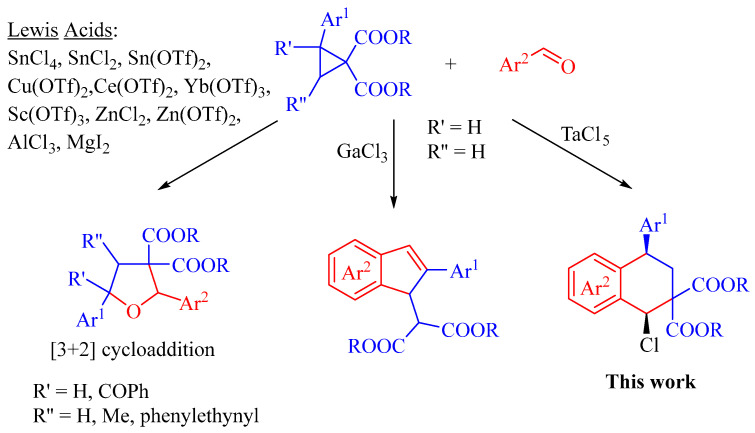
New type of DAC reactivity with aromatic aldehydes.

In this paper, we report a new type of 2-arylcyclopropane-1,1-dicarboxylate reactivity with aromatic aldehydes to produce tetrahydronaphthalene (tetraline) derivatives. This approach is based on the use of TaCl_5_ and is demonstrated in Scheme 1. The tetrahydronaphthalene moiety is a key structural motif in molecules of interest that have diverse biological properties, including anticancer, antimicrobial and antiviral activity [21,22].

## 2. Results and Discussion

**Scheme 2:**
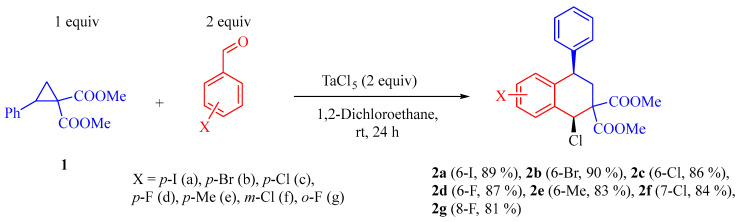
The reaction of dimethyl 2-phenylcyclopropane-1,1-dicarboxylate 1 with aromatic aldehydes and TaCl_5_.

Now, we report that the reaction of dimethyl 2-phenylcyclopropane-1,1-dicarboxylate **1** with **2** equivalents each of aromatic aldehydes and TaCl_5_ in 1,2-dichloroethane at 23 °C for 24 h after hydrolysis gives substituted 4-phenyl-3,4-dihydronaphtalene-2,2(1*H*)-dicarboxylates **2a–g** in good yield (Scheme 2). Two methods of loading reagents were used: (a) a glass reactor was charged with 4 mL of 1,2-dichloroethane, 1 mmol each of dimethyl 2-phenylcyclopropane-1,1-dicarboxylate and TaCl_5_ at 0 °C; then, after 10 min, 2 mmol of aromatic aldehyde and 1 mmol of TaCl_5_ were added to the reaction mixture at room temperature; (b) a glass reactor was charged with 4 mL of 1,2-dichloroethane, 1 mmol of dimethyl 2-phenylcyclopropane-1,1-dicarboxylate, 2 mmol of aromatic aldehyde and 2 mmol of TaCl_5_ at 0 °C. The yields of the reaction products did not vary significantly when the method of adding the reagents was changed.

**Figure 1:**
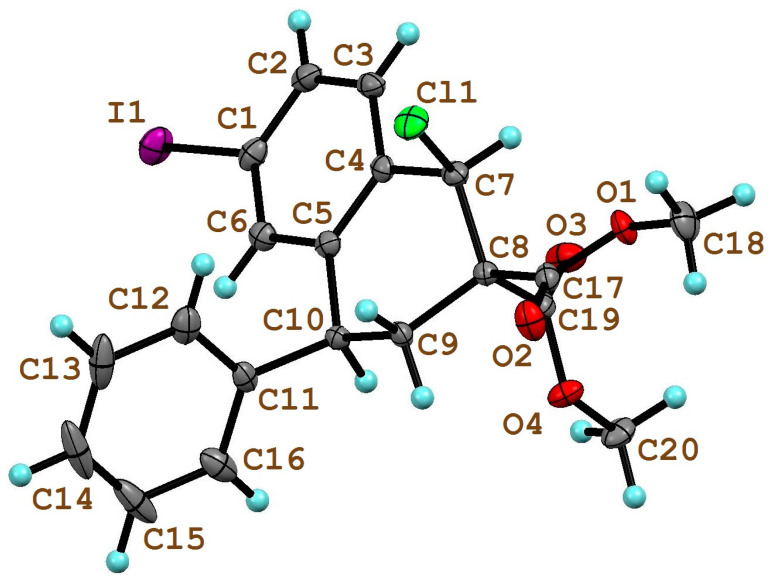
X-ray structure of substituted tetrahydronaphthalene **2a**.

The molecular structure of substituted 4-phenyl-3,4-dihydronaphtalene-2,2(1*H*)-dicarboxylates **2a–g** was identified by one-dimensional (^1^H, ^13^C) and two-dimensional (HSQS, HMBC, COSY, NOESY) NMR spectral methods (see Supplementary Materials). The X-ray diffraction analysis performed on substituted tetrahydronaphthalene **2a** allowed for the unambiguous attribution of relative stereochemistry (Figure 1).

TaCl_5_ has proven to be a unique Lewis acid that facilitates a new mode of interaction between 2-arylcyclopropane-1,1-dicarboxylates and aromatic aldehydes. To our knowledge, the formation of tetrahydronaphthalenes in these reactions has not been previously reported. All previously used Lewis acids in the reaction between 2-arylcyclopropane-1,1-dicarboxylates and aromatic aldehydes resulted in the formation of products with different structures (see Scheme 1).

**Scheme 3:**
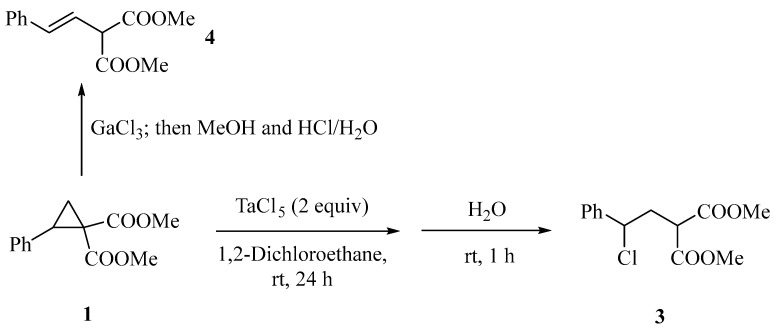
The formation of dimethyl 2-(2-chloro-2-phenylethyl)malonate **3**.

To better understand the reaction mechanism, we studied the reaction between dimethyl 2-phenylcyclopropane-1,1-dicarboxylate **1** and **2** equivalents of TaCl_5_ in 1,2-dichloroethane at room temperature. The reaction mixture was stirred for 18 h and quenched with water. It was found that, as a result of the reaction, all of the original DAC **1** had been converted to dimethyl 2-(2-chloro-2-phenylethyl)malonate **3**, with a yield of 81% (Scheme 3).

It is known that when dimethyl 2-arylcyclopropane-1,1-dicarboxylates **1** react with SnCl_4_ or TiCl_4_ without aldehyde presence, a fast cyclopropane ring opening occurs, and a similar chlorinated product is formed after quenching with water or methanol [23]. At the same time, in the presence of GaCl_3_, the reaction yields *b*-styryl malonate **4** upon quenching [20]. In this reaction, the behavior of TaCl_5_ is similar to that of TiCl_4_ and SnCl_4_. However, in the reaction of 2-arylcyclopropane-1,1-dicarboxylates **1** with aromatic aldehydes, the formation of substituted tetrahydronaphthalenes **2** was not observed under the influence of the two last Lewis acids, as it was when using TaCl_5_. This suggests that the cause of the unusual course of the reaction between 2-arylcyclopropane-1,1-dicarboxylates **1** and aromatic aldehydes in the presence of TaCl_5_ may be due to the nature of the interaction between aromatic aldehydes and TaCl_5_. Indeed, TaCl_5_ has a strong affinity for the oxygen atoms in carbonyl and carboxyl groups, acting as a powerful Lewis acid [24]. 

**Scheme 4:**
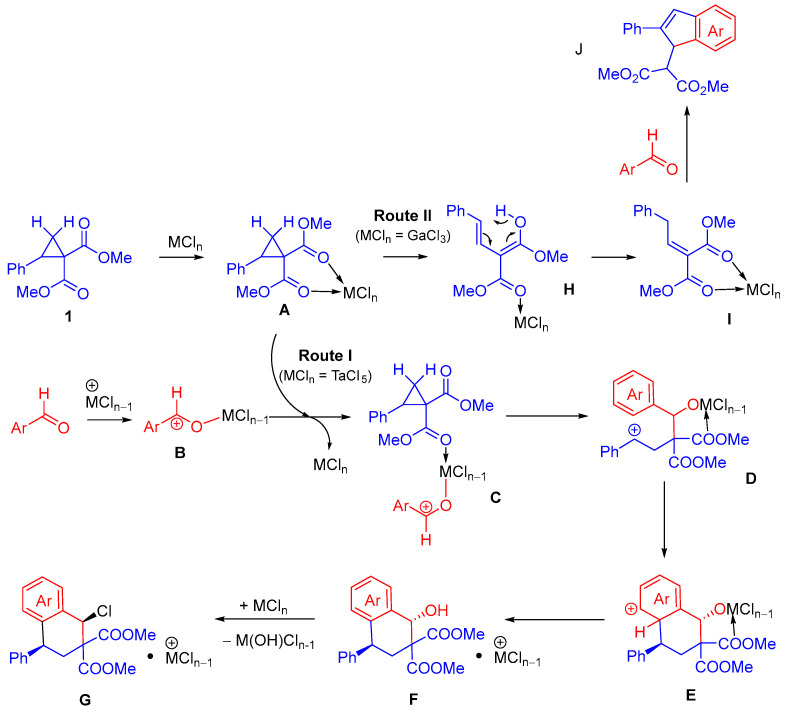
Plausible reaction mechanism.

We hypothesized that the key factor in determining the unique course of the reaction along a route I (Scheme 4) is the formation of an intermediate **B**, which is an active carbocation. This intermediate is formed through the interaction between an aromatic aldehyde and TaCl_5_ in ionic form. According to the proposed mechanism, the subsequent reaction of the intermediate **B** with complex **A** (complex between 2-arylcyclopropane-1,1-dicarboxylate **1** and TaCl_5_) results in the formation of an intermediate **C**, in which a tantalum atom is bonded to OCHAr and carboxylate groups. The subsequent electrophilic attack by the positively charged methine carbon atom on the quaternary carbon atom in the cyclopropane ring causes its opening and the formation of the carbocationic intermediate **D**. Next, the reaction of electrophilic substitution into an aromatic ring with the participation of an aryl substituent derived from the aromatic aldehyde results in the sequential formation of cyclic intermediates **E** and **F**. At the final stage of the reaction, the hydroxyl group is substituted by a chlorine atom through the S_N_2 mechanism, under the action of tantalum(V) chloride, to form intermediate **G**. The reaction of 2-arylcyclopropane-1,1-dicarboxylates **1** with aromatic aldehydes in the presence of GaCl_3_, which proceeds along a route II, has been previously described in detail [25]. 

**Figure 2:**
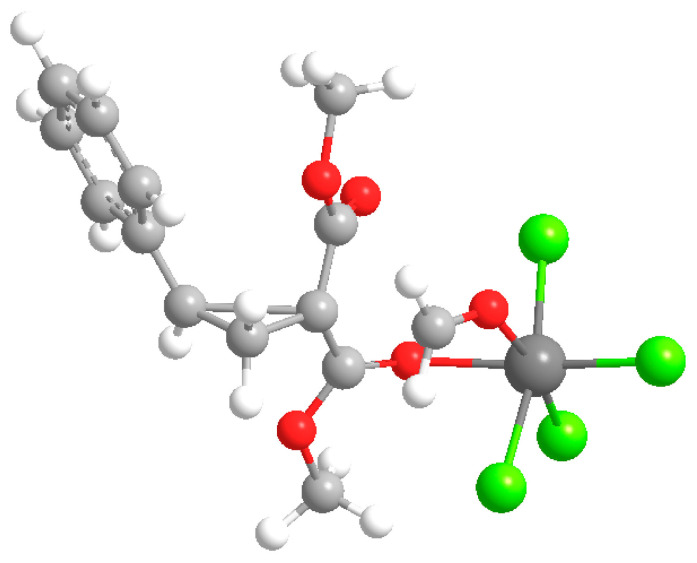
The transition state for the reaction of formaldehyde with dimethyl 1-phenylcyclopropane-1,1-dicarboxylate in complex with TaCl_4_^+^.

The key step in the proposed mechanism is the conversion of the intermediate **C** to **D**, during which the cyclopropane ring opens. According to the results of quantum chemical modeling using the B3LYP/6-31G(d)/LanL2DZ method, the activation barrier for the reaction between formaldehyde and dimethyl 1-phenylcyclopropane-1,1-dicarboxylate in complex with TaCl_4_^+^ was found to be 14.56 kcal/mol. The structure of the transition state is shown in Figure 2.

According to Scheme 4, the relative stereochemistry of carbon atoms in the cyclohexane ring is determined during the formation of the intermediate **E**. The large steric bulk of the aryl group and the tantalum-containing unit favor their *trans* location in the transition state. The subsequent S_N_2 substitution of the oxygen atom with a chlorine atom under the action of TaCl_5_ results in a reversal of the configuration of the corresponding carbon atom, forming a reaction product **G** with *cis*-oriented aryl and chlorine substituents on the cyclohexane ring.

As mentioned above, in the absence of aromatic aldehydes, dimethyl 2-phenylcyclopropane-1,1-dicarboxylate **1** reacts with **2** equivalents of TaCl_5_ to form dimethyl 2-(2-chloro-2-phenylethyl)malonate **3**. We found that the reaction proceeds in a similar manner in the presence of *p*-methoxybenzaldehyde. It is possible that the presence of an electron-donating substituent in the aromatic ring decreases the electrophilicity of the intermediate **B**, and the pathway of the reaction proceeds along route II (Scheme 4). Probably for the same reason, aliphatic aldehydes do not exhibit activity in the reaction under study.

So, let us take a look at the pathways of the reaction we are studying (Scheme 4). The first pathway (route I in Scheme 4) is carried out using TaCl_5_ as a Lewis acid, and it leads to the formation of substituted tetrahydronaphthalenes. When GaCl_3_ is used as a Lewis acid, a second pathway (route II in Scheme 4) is realized. Obviously, the main difference between these Lewis acids is their acidity. The B3LYP/6-31G(d)/LanL2DZ method was used to calculate the geometric parameters of the complexes TaCl_5_, TiCl_4_, SnCl_4_, ZnCl_2_ and GaCl_3_ with methyl acetate (MeC(OMe)=O→MX_n_ complex). The carbonyl bond length decreases in the order of the following complexes: Ester–GaCl_3_ (1.246 Å) > Ester–TaCl_5_ (1.244 Å) > Ester–ZnCl_2_ (1.239 Å) > Ester–SnCl_4_ (1.238 Å) > Ester–TiCl_4_ (1.236 Å), which indicates the decreasing strength of the Lewis acid towards the carboxyl group in the sequence GaCl_3_–TaCl_5_–ZnCl_2_–SnCl_4_–TiCl_4_. We recently demonstrated the effectiveness of TaCl_5_ in activating carboxylic acid esters for the amidation reaction with amines [26,27]. Thus, if the Lewis acid is sufficiently strong, it promotes the rapid rearrangement of DAC **1** to intermediate **H** (route II). If the strength of the Lewis acid is not sufficient for this rearrangement, then the intermediate **A** acts as a nucleophile and undergoes electrophilic attack by the aldehyde complex containing the Lewis acid (route I). If the latter complex is not sufficiently electrophilic to react with DAC **1**, the formation of substituted tetrahydrofurans may occur. 

The main method for the synthesis of substituted 3,4-dihydronaphthalene-2,2(1*H*)-dicarboxylates is the intramolecular Lewis acid-catalyzed cyclization of malonates substituted at the 2,2-position with arylmethyl and unsaturated (4-bromobut-2-en-yl [28], propargyl [29], homoallenyl [30] and 2-alkenyl [31,32]) substituents. InCl_3_, (NHC)GaX_3_/AgSbF_6_, Fe(III) complex, Pd(OAc)_2_ and Hg(OTf)_2_ have been used as Lewis acids. None of the known methods lead to the production of tetrahydronaphthalenes that are similar in structure to compounds **2**. The presence of a chlorine atom on the tetrahydronaphthalene ring greatly expands the range of possible chemical transformations that can be carried out further, including dechlorination reactions. For example, these reactions can be facilitated by the use of frustrated Lewis acid–base pairs [33]. A significant positive aspect of this transformation is the high level of stereoselectivity in the reaction, which leads to the production of tetrahydronaphthalenes with a *cis* arrangement of the aryl and chlorine substituents in the cyclohexene moiety. 

## 3. Materials and Methods

### 3.1. General Information

**Figure 3:**
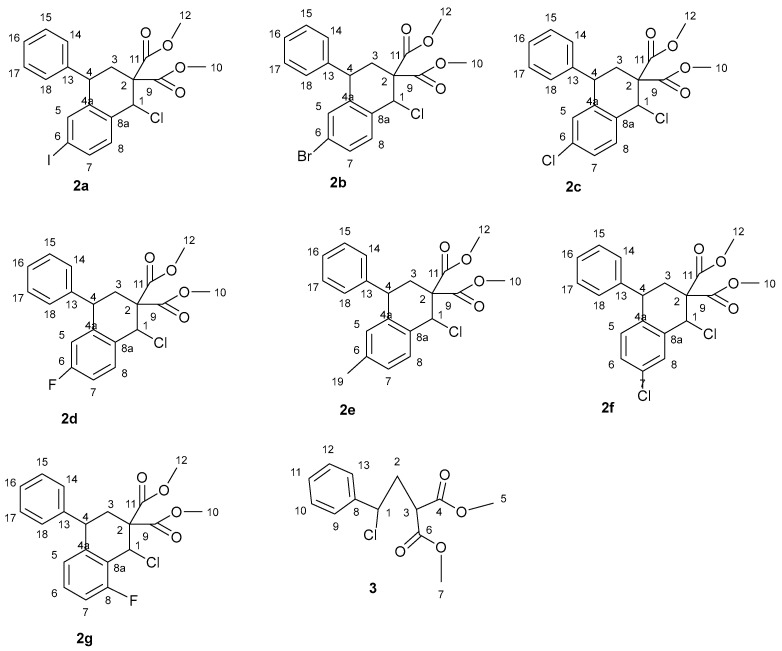
The numbering of atoms in the ^13^C and ^1^H NMR spectra of the compounds **2a–g**, **3**.

Chromatographic analysis was conducted on a Shimadzu GC-9A instrument (Shimadzu Corp., Kyoto, Japan) using a 2000 × 2 mm column with the SE-30 stationary phase (5%) on Chromaton N-AW-HMDS (0.125–0.160 mm) and helium as the carrier gas at a flow rate of 30 mL/min. The temperature was programmed from 50 to 300 °C at a rate of 8 degrees per minute. Nuclear magnetic resonance spectroscopy was conducted using a Bruker Avance 500 instrument (Bruker Corp., Bremen, Germany). The ^1^H nuclear magnetic resonance (NMR) spectra were recorded at a frequency of 500 megahertz (MHz), and the 13C-{1H} NMR spectra were collected at 125 MHz using CDCl_3_ as the solvent. The numbering of atoms in the ^13^C-{^1^H} and ^1^H NMR spectra of the compounds **2a–g**, **3** is shown in Figure 3. Elemental analysis was conducted using a Carlo Erba CHN 1106 elemental analyzer (Carlo Erba, Milan, Italy). Mass spectra were acquired using a Finnigan 4021 device (Finnigan-Mat. Co., San Jose, CA, USA). The yields were calculated from the isolated amount of 4-phenyl-3,4-dihydronaphtalene-2,2(1*H*)-dicarboxylate obtained from starting dimethyl 2-phenylcyclopropane-1,1-dicarboxylate and benzaldehyde derivative. Commercially available 4-iodobenzaldehyde, 4-bromobenzaldehyde, 4-chlorobenzaldehyde, 4-fluorobenzaldehyde, 4-methylbenzaldehyde, tantalum(V) chloride and 1,2-dichloroethane were obtained from Sigma-Aldrich (Merck Life Science LLC, An affiliate of Merck KGaA, Darmstadt, Germany) or Acros (Thermo Fisher Scientific GmbH, Dreieich, Germany). Dimethyl 2-phenylcyclopropane-1,1-dicarboxylate was prepared in three stages using the following methods. At the first stage, 1-phenyl-1,2-ethanediol was obtained based on the oxidation reaction of styrene with *m*-chloroperbenzoic acid [34]. At the second stage, based on the reaction of 1-phenyl-1,2-ethanediol with methanesulfonyl chloride, bis(methanesulfonate) ester was obtained [35]. Next, the target dimethyl 2-phenylcyclopropane-1,1-dicarboxylate was obtained by reacting bis(methanesulfonate) ester with dimethyl malonate [36]. CCDC 2355172 contains the supplementary crystallographic data for this paper. These data can be obtained free of charge from The Cambridge Crystallographic Data Centre via http://www.ccdc.cam.ac.uk (accessed on 31 May 2024). All quantum chemical calculations were performed using the B3LYP/6-31G(d)/LanL2DZ basis set as implemented in Gaussian 09 software [37].

### 3.2. Preparation of 4-Phenyl-3,4-dihydronaphtalene-2,2(1H)-dicarboxylate ***2a–g*** via Reaction of Dimethyl 2-Phenylcyclopropane-1,1-dicarboxylate and Benzaldehyde Derivative

#### 3.2.1. Dimethyl 1-Chloro-6-iodo-4-phenyl-3,4-dihydronaphtalene-2,2(1*H*)-dicarboxylate (**2a**) (Typical Procedure)

Dimethyl 2-phenylcyclopropane-1,1-dicarboxylate (117 mg, 0,5 mmol) was placed in a 50 mL reaction flask, under an argon atmosphere. A 50 mL glass reactor equipped with a magnetic stirrer under a dry argon atmosphere at 0 °C was charged under stirring with 2-phenylcyclopropane-1,1-dicarboxylate (234 mg, 1 mmol), TaCl_5_ (358 mg, 1 mmol) and 1,2-dichloroethane (4 mL). The reaction mixture was stirred at 0 °C for 10 min. 4-iodobenzaldehyde (464 mg, 2 mmol) and TaCl_5_ (358 mg, 1 mmol) were added to the reaction mixture at room temperature successively, and the resulting mixture was stirred at room temperature for 24 h. Then, the reaction mixture was diluted with dichloromethane (20 mL), and distilled water (15 mL) was added dropwise, while the reaction flask was cooled in an ice bath. The aqueous layer was extracted with dichloromethane (3 × 20 mL). The combined organic layers were washed with brine (20 mL), dried over anhydrous MgSO_4_. The reaction mixture was filtered through a filter paper and concentrated in vacuo to give a crude product that was purified by column chromatography (petroleum ether–ethyl acetate = 6:1) to afford **2a** (432 mg, 89%) as colorless crystals: mp, 148–150 °C. ^1^H NMR (500 MHz, CDCl_3_): δ = 2.76 (t, *J* = 3.1 Hz, 2H, C(3)H_2_), 3.76 (s, 3H, C(10)H_3_), 3.85 (s, 3H, C(12)H_3_), 3.86–3.88 (m, 1H, C(4)H), 5.79 (s, 1H, C(1)H), 7.15–7.17 (m, 2H, C(5,8)H), 7.21 (d, *J* = 7.3 Hz, 2H, C(14,18)H), 7.28–7.33 (m, 1H, C(16)H), 7.38 (t, *J* = 7.4 Hz, 3H, C(15,17)H), 7.58 (d, *J* = 8.3 Hz, 1H, C(7)H). ^13^C NMR (500 MHz, CDCl_3_): δ = 32.15 (C(3)), 42.93 (C(4)), 53.27 (C(12)), 53.36 (C(10)), 58.19 (C(1)), 59.32 (C(2)), 95.05 (C(6)), 127.24 (C(16)), 128.80 (C(14,18)), 128.98 (C(15,17)), 131.53 (C(8)), 135.06 (C(8a)), 136.33 (C(7)),138.69 (C(5)), 139.74 (C(4a)), 144.12 (C(13)), 168.08 (C(11)), 168.58 (C(9)). MS (EI): *m*/*z*, % = 449 (2) [M-Cl]^+^, 448 (7), 389 (22), 388 (22), 357 (5), 330 (6), 262 (12), 218 (13), 203 (22), 202 (25), 127 (7), 59 (100). Anal. calcd for C_20_H_18_ClIO_4_, (%): C, 49.56; H, 3.74; Found, %: C, 49.8; H, 3.9.

#### 3.2.2. Crystal Data for Dimethyl 1-Chloro-6-iodo-4-phenyl-3,4-dihydronaphtalene-2,2(1*H*)-dicarboxylate (**2a**)

Crystals are monoclinic (space group P2_1_/n), chemical composition C_20_H_18_ClIO_4_, a = 14.4692(4), b = 13.0551(5), c = 21.7457(8) Å, α = 90, β = 109.3060(10), γ = 90 °, V = 3876.7(2) Å³, Z = 8, d_calc_ = 1.661 g·cm^−3^, F(000) = 1920, M = 484.69. A single crystal (yellow irregular-shaped, dimensions 0.24 mm × 0.29 mm × 0.37 mm) was selected, and the intensities of 41098 reflections were measured with a Bruker APEX-II CCD diffractometer at 120 K (ϕ and ω scans, sealed tube, λ[MoKα] = 0.71073 Å, μ = 1.812 mm^−1^, 2θ_max_ = 61.066°). After the merging of equivalents and absorption correction, 11519 independent reflections (R_int_ = 0.0293) were used for the structure solution and refinement. The structure was solved by the dual space method and refined by the full-matrix technique against F^2^ in anisotropic approximation. The positions of hydrogen atoms in methyl and methylene groups were calculated geometrically and refined in rigid body approximation. Final R factors: R_1_ = 0.0242, (9285 reflections with I > σ(I)), wR_2_ = 0.0586 (all reflections), GOF = 1.103. The structure was solved with the ShelXT [38] program and refined with the ShelXL [39] program (version 2019/2). Molecular graphics were drawn using the OLEX2 [40] program (version 1.5). The dataset for **2a** was measured in Centre for molecular composition studies of INEOS RAS. CCDC 2355172 contains the supplementary crystallographic data for **2a**. These data can be obtained free of charge from The Cambridge Crystallographic Data Centre via https://www.ccdc.cam.ac.uk/structures (accessed on 31 May 2024).

#### 3.2.3. Dimethyl 6-Bromo-1-chloro-4-phenyl-3,4-dihydronaphtalene-2,2(1*H*)-dicarboxylate (**2b**)

Using the procedure described above, 370 mg of 4-bromobenzaldehyde (2 mmol) gave a crude product that was purified by column chromatography (petroleum ether–ethyl acetate = 6:1) to afford **2b** (394 mg, 90%) as colorless crystals: mp, 150–152 °C.

^1^H NMR (500 MHz, CDCl_3_): δ = 2.75–2.79 (m, 2H, C(3)H_2_), 3.76 (s, 3H, C(10)H_3_), 3.85 (s, 3H, C(12)H_3_), 3.87–3.89 (m, 1H, C(4)H), 5.79 (s, 1H, C(1)H), 6.96 (br.s, 2H, C(5,7)H), 7.21 (d, *J* = 7.2 Hz, 2H, C(14,18)H), 7.28–7.33 (m, 2H, C(8,16)H), 7.38 (t, *J* = 7.4 Hz, 2H, C(15, 17)H). ^13^C NMR (500 MHz, CDCl_3_): δ = 32.09(C(3)), 43.10(C(4)), 53.27(C(12)), 53.34 (C(10)), 58.07(C(1)), 59.38 (C(2)), 122.99 (C(6)), 127.24 (C(16)), 128.81 (C(14,18)), 128.98 (C(15,17)), 130.48 (C(8)), 131.48 (C(7)), 134.37 (C(8a)), 139.69 (C(4a)), 144.07 (C(13)), 168.08 (C(11)), 168.59 (C(9)). MS (EI): *m*/*z*, % = 402 (15) [M-Cl]^+^, 401 (2), 400 (12), 344 (17), 343 (59), 342 (48), 341 (55), 340 (58), 311 (17), 282 (15), 262 (22), 218 (57), 203 (57), 202 (66), 59 (100). Anal. calcd for C_20_H_18_BrClO_4_, (%): C, 54.88; H, 4.15; Found, %: C, 55.0; H, 3.9.

#### 3.2.4. Dimethyl 1,6-Dichloro-4-phenyl-3,4-dihydronaphtalene-2,2(1*H*)-dicarboxylate (**2c**)

Using the procedure described above, 282 mg of 4-chlorobenzaldehyde (2 mmol) gave a crude product that was purified by column chromatography (petroleum ether–ethyl acetate = 6:1) to afford **2c** (338 mg, 86%) as colorless crystals: mp, 153–155 °C. ^1^H NMR (500 MHz, CDCl_3_): δ = 2.78–2.82 (m, 2H, C(3)H_2_), 3.76 (s, 3H, C(10)H_3_), 3.85 (s, 3H, C(12)H_3_), 3.87–3.89 (m, 1H, C(4)H), 5.82 (s, 1H, C(1)H), 6.80 (s, 1H, C(5)H), 7.22 (d, *J* = 7.3 Hz, 2H, C(14,18)H), 7.28–7.33 (m, 2H, C(8,16)H), 7.36–7.38 (m, 3H, C(7,15,17)H). ^13^C NMR (500 MHz, CDCl_3_): δ = 32.07 (C(3)), 43.15 (C(4)), 53.26 (C(12)), 53.33 (C(10)), 58.07 (C(1)), 59.43 (C(2)), 127.24 (C(16)), 127.59 (C(8)), 128.82 (C(14,18)), 128.98 (C(15,17)), 129.67 (C(5)), 131.29 (C(7)), 133.86 (C(6)), 134.69 (C(8a)), 139.42 (C(4a)), 144.09 (C(13)), 168.09 (C(11)), 168.59 (C(9)). MS (EI): *m*/*z*, % = 358 (4) [M-Cl]^+^, 357 (3), 356 (15), 297 (78), 296 (61), 267 (13), 265 (23), 238 (18), 218 (30), 217 (25), 204 (19), 203 (41), 202 (47), 59 (100). Anal. calcd for C_20_H_18_Cl_2_O_4_, (%): C, 61.08; H, 4.61; Found, %: C, 61.1; H, 4.4.

#### 3.2.5. Dimethyl 1-Chloro-6-fluoro-4-phenyl-3,4-dihydronaphtalene-2,2(1*H*)-dicarboxylate (**2d**)

Using the procedure described above, 248 mg of 4-fluorobenzaldehyde (2 mmol) gave a crude product that was purified by column chromatography (petroleum ether–ethyl acetate = 6:1) to afford **2d** (328 mg, 87%) as colorless crystals: mp, 122–124 °C. ^1^H NMR (500 MHz, CDCl_3_): δ = 2.77–2.79 (m, 2H, C(3)H_2_), 3.76 (s, 3H, C(10H_3_), 3.85 (s, 3H, C(12)H_3_), 3.88–3.89 (m, 1H, C(4)H), 5.85 (s, 1H, C(1)H), 6.49 (d, *J* = 9.4 Hz, 1H, C(5)H), 6.96 (d, *J* = 8.0 Hz, 1H, C(7)H), 7.22 (d, *J* = 7.5 Hz, 2H, C(14,18)H), 7.28–7.32 (m, 1H, C(16)H), 7.36–7.43 (m, 3H, C(8,15,17)H). ^13^C NMR (500 MHz, CDCl_3_): δ = 31.91 (C(3)), 43.33 (C(4)), 53.24 (C(12)), 53.29 (C(10)), 58.22 (C(1)), 59.56 (C(2)), 114.83 (d, *J* = 22.2 Hz, C(7)), 116.1 (d, *J* = 22.1 Hz, C(5)), 128.81 (C(14,18)), 128.92 (C(15,17)), 131.19 (C(8)), 131.80 (d, *J* = 8.3 Hz, C(8a)), 140.10 (d, *J* = 7.7 Hz, C(4a)), 144.17 (C(13)), 161.59, 163.57 (C(6)), 168.18 (C(11)), 168.62 (C(9)). MS (EI): *m*/*z*, % = 341 (5) [M-Cl]^+^, 340 (17), 281 (81), 280 (67), 249 (30), 222 (35), 221 (37), 220 (34), 203 (12), 202 (20), 196 (14), 173 (7), 146 (19), 145 (19), 125 (14), 59 (100). Anal. calcd for C_20_H_18_ClFO_4_, (%): C, 63.75; H, 4.82; Found, %: C, 64.1; H, 4.8.

#### 3.2.6. Dimethyl 1-Chloro-6-methyl-4-phenyl-3,4-dihydronaphtalene-2,2(1*H*)-dicarboxylate (**2e**)

Using the procedure described above, 240 mg of 4-methylbenzaldehyde (2 mmol) gave a crude product that was purified by column chromatography (petroleum ether–ethyl acetate = 6:1) to afford **2e** (310 mg, 83%) as colorless crystals: mp, 134–136 °C. ^1^H NMR (500 MHz, CDCl_3_): δ = 2.19 (s, 3H, (C(19)H_3_), 2.77–2.79 (m, 2H, C(3)H_2_), 3.75 (s, 3H, C(10)H_3_), 3.85 (s, 3H, C(12)H_3_), 3.89 (t, *J* = 9.3 Hz, 1H, C(4)H), 5.86 (s, 1H, C(1)H), 6.61 (s, 1H, C(5)H), 7.07 (d, *J* = 7.8 Hz,1H, C(8)H), 7.24 (d, *J* = 7.4 Hz, 2H, C(14,18)H), 7.28–7.38 (m, 4H, C(7,15,16,17)H). ^13^C NMR (500 MHz, CDCl_3_): δ = 21.26 (C(19)), 32.52 (C(3)), 43.15 (C(4)), 53.16 (C(12)), 53.19 (C(10)), 58.07 (C(1)), 59.59 (C(2)), 126.83 (C(16)), 128.17 (C(8)), 128.73 (C(14,18)), 128.92 (C(15,17)), 129.82 (C(7)), 130.18 (C(5)), 132.35 (C(6)), 137.23 (C(8a)), 138.79 (C(4a)), 145.21 (C(13)), 168.45 (C(11)), 168.77 (C(9)). MS (EI): *m*/*z*, % = 337 (7) [M-Cl]^+^, 336 (31), 278 (25), 277 (100), 245 (66), 218 (48), 203 (32), 202 (36), 141 (19), 115 (14), 59 (100). Anal. calcd for C_21_H_21_ClO_4_, (%): C, 67.65; H, 5.68; Found, %: C, 67.5; H, 5.8.

#### 3.2.7. Dimethyl 1,7-Dichloro-4-phenyl-3,4-dihydronaphthalene-2,2(1*H*)-dicarboxylate (**2f**)

Using the procedure described above, 281 mg of 3-chlorobenzaldehyde (2 mmol) gave a crude product that was purified by column chromatography (petroleum ether–ethyl acetate = 6:1) to afford **2f** (330 mg, 84%) as colorless crystals: mp, 149–151 °C. ^1^H NMR (500 MHz, CDCl_3_): δ = 2.71–2.81 (m, 2H, C(3)H_2_), 3.77 (s, 3H, C(10)H_3_), 3.85 (s, 4H, C(4)H, C(12)H_3_), 5.77 (s, 1H, C(1)H), 6.75 (d, *J* =8.5 Hz, 1H, C(5)H), 7.12 (dd, *J* = 1.7 Hz, *J* = 8.5 Hz, 1H, C(6)H), 7.20 (d, *J* = 7.3 Hz, 2H, C(14,18)H), 7.28–7.31 (m, 1H, C(16)H), 7.35 (t, *J* = 7.1 Hz, 2H, C(15,17)H), 7.41 (s, 1H, C(8)H). ^13^C NMR (500 MHz, CDCl_3_): δ = 32.21 (C(3)), 42.81 (C(4)), 53.28 (C(12)), 53.34 (C(10)), 57.89 (C(1)), 59.39 (C(2)), 127.12 (C(16)), 128.79 (C(14,18)), 128.87 (C(15,17)), 129.13 (C(6)), 129.53 (C(8)), 131.33 (C(5)), 132.59 (C(7)), 135.98 (C(4a)), 136.94 (C(8a)), 144.47 (C(13)), 168.06 (C(11)), 168.61 (C(9)). MS (EI): *m*/*z*, % = 357 (6) [M-Cl]^+^, 356 (27), 324 (7), 297 (100), 296 (76), 265 (40), 238 (23), 218 (45), 217 (29), 203 (46), 202 (57), 101 (11), 59 (100). Anal. calcd for C_20_H_18_Cl_2_O_4_, (%): C, 61.08; H, 4.61; Found, %: C, 61.0; H, 4.5.

#### 3.2.8. Dimethyl 1-Chloro-8-fluoro-4-phenyl-3,4-dihydronaphthalene-2,2(1*H*)-dicarboxylate (**2g**)

Using the procedure described above, 248 mg of 2-fluorobenzaldehyde (2 mmol) gave a crude product that was purified by column chromatography (petroleum ether–ethyl acetate = 6:1) to afford **2g** (305 mg, 81%) as colorless crystals: mp, 144–146 °C. ^1^H NMR (500 MHz, CDCl_3_): δ = 2.79 (d, *J* = 9.7 Hz, 2H, C(3)H_2_), 3.76 (s, 3H, C(10)H_3_), 3.86 (s, 3H, C(12)H_3_), 3.91 (t, *J* = 9.7 Hz, 1H, C(4)H), 6.07 (s, 1H, C(1)H), 6.61 (d, *J* = 7.7 Hz, 1H, C(5)H), 6.97 (t, *J* = 8.9 Hz, 1H, C(7)H), 7.13–7.17 (q, *J* = 7.7 Hz, 1H, C(6)H), 7.22 (d, *J* = 7.3 Hz, 2H, C(14,18)H), 7.30 (t, *J* = 7.3 Hz, 1H, C(16)H), 7.36 (t, *J* = 7.3 Hz, 2H, C(15,17)H). ^13^C NMR (500 MHz, CDCl_3_): δ = 32.12 (C(3)), 43.14 (C(4)), 51.80 (d, *J* = 5.7 Hz, C(1)), 53.27 (C(12)), 53.31 (C(10)), 59.06 (C(2)), 113.38 (d, *J* = 20.9 Hz, C(7)), 123.83 (d, *J* = 14.7 Hz, C(8a)), 125.35 (d, *J* = 3.3 Hz, C(5)), 127.09 (C(16)), 128.82 (C(14,18)), 128.84 (C(15,17)), 129.78 (d, *J* = 9.1 Hz, C(6)), 139.75 (C(4a)), 144.50 (C(13)), 159.23, 161.22 (C(8)), 168.07 (C(11)), 168.55 (C(9)). MS (EI): *m*/*z*, % = 341 (4) [M-Cl]^+^, 340 (18), 281 (100), 280 (80), 249 (20), 237 (13), 222 (38), 221 (31), 202 (23), 146 (11), 59 (25). Anal. calcd for C_20_H_18_ClFO_4_, (%): C, 63.75; H, 4.82; Found, %: C, 63.3; H, 4.9.

#### 3.2.9. Dimethyl 2-(2-Chloro-2-phenylethyl)malonate (**3**) [21]

Using the procedure described but in the absence of a benzaldehyde derivative gave a crude product that was purified by column chromatography (petroleum ether–ethyl acetate = 6:1) to afford **4** (305 mg, 81%) as a colorless oil. ^1^H NMR (500 MHz, CDCl_3_): δ = 2.66 (t, *J* = 7.3 Hz, 2H, C(2)H_2_), 3.69 (t, *J* = 7.3 Hz, 1H, C(3)H), 3.77 (s, 3H, C(7)H_3_), 3.78 (s, 3H, C(5)H_3_), 4.97 (t, *J* = 7.3 Hz, 1H, C(1)H), 7.33–7.42 (m, 5H, C(9,10,11,12,13)H). ^13^C NMR (500 MHz, CDCl_3_): δ = 38.78 (C(2)), 49.41 (C(3)), 52.79 (C(5)), 52.81 (C(7)), 60.85 (C(1)), 126.92 (C(9,13)), 128.70 (C(11)), 128.80 (C(10,12)), 140.45 (C(8)), 168.99 (C(4)), 169.12 (C(6)). MS (EI): *m*/*z*, % = 271 (<1) [M]^+^, 270 (2), 171 (3), 132 (100), 115 (61), 100 (25), 59 (31).

## 4. Conclusions

In conclusion, we have developed a new reaction of arylcyclopropanedicarboxylates with aromatic aldehydes in the presence of TaCl_5_ as a route to substituted tetrahydronaphthalenes. The presence of a chlorine atom in the tetrahydronaphthalene ring significantly expands the range of potential chemical reactions to modify the obtained compounds. One major advantage of this reaction is its strong preference for a specific stereochemistry, which results in the formation of tetrahydronaphthalenes containing aryl and chlorine substituents in a *cis* configuration at the cyclohexene ring.

## Data Availability

The raw data supporting the conclusions of this article will be made available by the authors on request.

## Supplementary Materials

The following supporting information can be downloaded at https://www.mdpi.com/article/10.3390/molecules29122715/s1.
