# Supplementary material for: The TaCl5-Mediated Reaction of Dimethyl 2-Phenylcyclopropane-1,1-dicarboxylate with Aromatic Aldehydes as a Route to Substituted Tetrahydronaphthalenes
Source: Molecules. 2024 Jun 7;29(12):2715. doi: 10.3390/molecules29122715 (PMC11205635; doi:10.3390/molecules29122715)

$TaCl_5$-Mediated Reaction of Dimethyl 2-Phenylcyclopropane-1,1-dicarboxylate with Aromatic Aldehydes as a Route to Substituted Tetrahydronaphthalenes

Supporting information

General information

1.1. General information. Chromatographic analysis was conducted on a Shimadzu GC-9A instrument (Shimadzu Corporation, Tokio, Japan) using a 2000 × 2 mm column with the SE-30 stationary phase (5%) on Chromaton N-AW-HMDS (0.125–0.160 mm) and helium as the carrier gas at a flow rate of 30 mL/min. The temperature was programmed from 50 to 300 °C at a rate of 8 degrees per minute. Nuclear magnetic resonance spectroscopy was conducted using a Bruker Avance 500 instrument (Bruker Corporation, Bremen, Germany). The ^1H nuclear magnetic resonance (NMR) spectra were recorded at a frequency of 500 megahertz (MHz), and the 13C-{1H} NMR spectra were collected at 125 MHz using $CDCl_3$ as the solvent. Elemental analysis was conducted using a Carlo Erba CHN 1106 elemental analyzer (Carlo Erba, Milan, Italy). Mass spectra were acquired using a Finnigan 4021 device (Finnigan-Mat. Co., San Jose, USA). The yields were calculated from the isolated amount of 4-phenyl-3,4-dihydronaphtalene-2,2(1*H*)-dicarboxylate obtained from starting dimethyl 2-phenylcyclopropane-1,1-dicarboxylate and benzaldehyde derivative. Commercially available 4-iodobenzaldehyde, 4-bromobenzaldehyde, 4-chlorobenzaldehyde, 4-fluorobenzaldehyde, 4-methylbenzaldehyde, tantalum(V) chloride and 1,2-dichloroethane were obtained from Sigma-Aldrich (Merck Life Science LLC, An affiliate of Merck KGaA, Darmstadt, Germany) or Acros (Thermo Fisher Scientific GmbH, Dreieich, Germany). Dimethyl 2-phenylcyclopropane-1,1-dicarboxylate

was prepared in three stages using the following methods. At the first stage, 1-phenyl-1,2-ethanediol was obtained based on the oxidation reaction of styrene with *m*-chloroperbenzoic acid [1]. At the second stage, based on the reaction of 1-phenyl-1,2-ethanediol with methanesulfonyl chloride, bis(methanesulfonate) ester was obtained [2]. Next, the target dimethyl 2-phenylcyclopropane-1,1-dicarboxylate was obtained by reacting bis(methanesulfonate) ester with dimethyl malonate [3]. CCDC 2355172 contains the supplementary crystallographic data for this paper. These data can be obtained free of charge from The Cambridge Crystallographic Data Centre via http://www.ccdc.cam.ac.uk (accessed on 31 May 2024). All quantum chemical calculations were performed using the B3LYP/6-31G(d)/LanL2DZ basis set as implemented in Gaussian 09 software [4].

1.2. Preparation of 4-phenyl-3,4-dihydronaphtalene-2,2(1H)-dicarboxylate ***2a-g*** *via reaction dimethyl 2-phenylcyclopropane-1,1-dicarboxylate and benzaldehyde derivative.*

*1.2.1. Dimethyl 1-chloro-6-iodo-4-phenyl-3,4-dihydronaphtalene-2,2(1H)-dicarboxylate (**2a**) (Typical Procedure).* Dimethyl 2-phenylcyclopropane-1,1-dicarboxylate (117 mg, 0,5 mmol) was placed in a 50 mL reaction flask, under an argon atmosphere. A 50 mL glass reactor equipped with a magnetic stirrer under a dry argon atmosphere at 0 °C was charged under stirring with 2-phenylcyclopropane-1,1-dicarboxylate (234 mg, 1 mmol), $TaCl_5$ (358 mg, 1 mmol) and 1,2-dichloroethane (4 mL). The reaction mixture was stirred at 0 °C for 10 min. 4-iodobenzaldehyde (464 mg, 2 mmol) and $TaCl_5$ (358 mg, 1 mmol) were added to the reaction mixture at room temperature successively, and the resulting mixture was stirred at room temperature for 24 h. Then, the reaction mixture was diluted with dichloromethane (20 mL), and distilled water (15 mL) was added dropwise, while the reaction flask was cooled in an ice bath. The aqueous layer was extracted with dichloromethane (3 × 20 mL). The combined organic layers were washed with

brine (20 mL), dried over anhydrous $MgSO_4$. The reaction mixture was filtered through a filter paper and concentrated in vacuo to give a crude product that was purified by column chromatography (petroleum ether–ethyl acetate = 6:1) to afford **2a** (432 mg, 89%) as colorless crystals: mp, 148-150 ^oC. ^1H NMR (500 MHz, $CDCl_3$): δ = 2.76 (t, *J* = 3.1 Hz, 2H, C(3)H_2), 3.76 (s, 3H, C(10)H_3), 3.85 (s, 3H, C(12)H_3), 3.86 – 3.88 (m, 1H, C(4)H), 5.79 (s, 1H, C(1)H), 7.15 – 7.17 (m, 2H, C(5,8)H), 7.21 (d, *J* = 7.3 Hz, 2H, C(14,18)H), 7.28 – 7.33 (m, 1H, C(16)H), 7.38 (t, *J* = 7.4 Hz, 3H, C(15,17)H), 7.58 (d, *J* = 8.3 Hz, 1H, C(7)H). ^{13}C NMR (500 MHz, $CDCl_3$): δ = 32.15 (C(3)), 42.93 (C(4)), 53.27 (C(12)), 53.36 (C(10)), 58.19 (C(1)), 59.32 (C(2)), 95.05 (C(6)), 127.24 (C(16)), 128.80 (C(14,18)), 128.98 (C(15,17)), 131.53 (C(8)), 135.06 (C(8a)), 136.33 (C(7)),138.69 (C(5)), 139.74 (C(4a)), 144.12 (C(13)), 168.08 (C(11)), 168.58 (C(9)). MS (EI): m/z, % = 449 (2) $[M-Cl]^+$, 448 (7), 389 (22), 388 (22), 357 (5), 330 (6), 262 (12), 218 (13), 203 (22), 202 (25), 127 (7), 59 (100). Anal. calcd for $C_{20}H_{18}ClIO_4$, (%): C, 49.56; H, 3.74; Found, %: C, 49.8; H, 3.9.

1.2.2. Crystal data for dimethyl 1-chloro-6-iodo-4-phenyl-3,4-dihydronaphtalene-2,2(1H)-dicarboxylate ***(2a):*** Crystals are monoclinic (space group $P2_1/n$), chemical composition $C_{20}H_{18}ClIO_4$, a = 14.4692(4), b = 13.0551(5), c = 21.7457(8) Å, α = 90, β = 109.3060(10), γ = 90 °, V = 3876.7(2) $Å^3$, Z = 8, d_{calc} = 1.661 g·cm^{-3}, F(000) = 1920, M = 484.69. A single crystal (yellow irregular-shaped, dimensions 0.24 mm × 0.29 mm × 0.37 mm) was selected, and the intensities of 41098 reflections were measured with a Bruker APEX-II CCD diffractometer at 120K (ϕ and ω scans, sealed tube, λ[MoKα] = 0.71073 Å, μ = 1.812 mm^{-1}, $2\theta_{max}$ = 61.066°). After the merging of equivalents and absorption correction, 11519 independent reflections (R_{int} = 0.0293) were used for the structure solution and refinement. The structure was solved by the dual space method and refined by the full-matrix technique against F^2 in anisotropic approximation. The positions of hydrogen atoms in methyl and methylene groups were calculated geometrically and refined in rigid body

approximation. Final R factors: R_1 = 0.0242, (9285 reflections with I > σ(I)), wR_2 = 0.0586 (all reflections), GOF = 1.103. The structure was solved with the ShelXT [38] program and refined with the ShelXL [39] program (version 2019/2). Molecular graphics were drawn using the OLEX2 [40] program (version 1.5). The dataset for **2a** was measured in Centre for molecular composition studies of INEOS RAS. CCDC 2355172 contains the supplementary crystallographic data for **2a**. These data can be obtained free of charge from The Cambridge Crystallographic Data Centre via https://www.ccdc.cam.ac.uk/structures (accessed on 31 May 2024).

*1.2.3. Dimethyl 6-bromo-1-chloro-4-phenyl-3,4-dihydronaphtalene-2,2(1H)-dicarboxylate (**2b**).* Using the procedure described above 370 mg of 4-bromobenzaldehyde (2 mmol) gave crude product that was purified by column chromatography (petroleum ether : ethyl acetate = 6 : 1) to afford **2b** (394 mg, 90%) as colorless crystals: mp, 150-152 °C. ^{1}H NMR (500 MHz, $CDCl_3$): δ = 2.75 – 2.79 (m, 2H, C(3)H_2), 3.76 (s, 3H, C(10)H_3), 3.85 (s, 3H, C(12)H_3), 3.87 – 3.89 (m, 1H, C(4)H), 5.79 (s, 1H, C(1)H), 6.96 (br.s, 2H, C(5,7)H), 7.21 (d, *J* = 7.2 Hz, 2H, C(14,18)H), 7.28 – 7.33 (m, 2H, C(8,16)H), 7.38 (t, *J* = 7.4 Hz, 2H, C(15, 17)H). ^{13}C NMR (500 MHz, $CDCl_3$): δ = 32.09(C(3)), 43.10(C(4)), 53.27(C(12)), 53.34 (C(10)), 58.07(C(1)), 59.38 (C(2)), 122.99 (C(6)), 127.24 (C(16)), 128.81 (C(14,18)), 128.98 (C(15,17)), 130.48 (C(8)), 131.48 (C(7)), 134.37 (C(8a)), 139.69 (C(4a)), 144.07 (C(13)), 168.08 (C(11)), 168.59 (C(9)). MS (EI): m/z, % = 402 (15) $[M-Cl]^+$, 401 (2), 400 (12), 344 (17), 343 (59), 342 (48), 341 (55), 340 (58), 311 (17), 282 (15), 262 (22), 218 (57), 203 (57), 202 (66), 59 (100). Anal. calcd for $C_{20}H_{18}BrClO_4$, (%): C, 54.88; H, 4.15; Found, %: C, 55.0; H, 3.9.

*1.2.4. Dimethyl 1,6-dichloro-4-phenyl-3,4-dihydronaphtalene-2,2(1H)-dicarboxylate (**2c**).* Using the procedure described above 282 mg of 4-chlorobenzaldehyde (2 mmol) gave crude product that was purified by column chromatography (petroleum ether : ethyl acetate = 6 : 1) to afford **2c** (338 mg, 86%) as colorless crystals: mp, 153-

155 °C. ^{1}H NMR (500 MHz, $CDCl_3$): δ = 2.78 – 2.82 (m, 2H, C(3)H_2), 3.76 (s, 3H, C(10)H_3), 3.85 (s, 3H, C(12)H_3), 3.87 – 3.89 (m, 1H, C(4)H), 5.82 (s, 1H, C(1)H), 6.80 (s, 1H, C(5)H), 7.22 (d, *J* = 7.3 Hz, 2H, C(14,18)H), 7.28 – 7.33 (m, 2H, C(8,16)H), 7.36 – 7.38 (m, 3H, C(7,15,17)H). ^{13}C NMR (500 MHz, $CDCl_3$): δ = 32.07 (C(3)), 43.15 (C(4)), 53.26 (C(12)), 53.33 (C(10)), 58.07 (C(1)), 59.43 (C(2)), 127.24 (C(16)), 127.59 (C(8)), 128.82 (C(14,18)), 128.98 (C(15,17)), 129.67 (C(5)), 131.29 (C(7)), 133.86 (C(6)), 134.69 (C(8a)), 139.42 (C(4a)), 144.09 (C(13)), 168.09 (C(11)), 168.59 (C(9)). MS (EI): m/z, % = 358 (4) $[M-Cl]^+$, 357 (3), 356 (15), 297 (78), 296 (61), 267 (13), 265 (23), 238 (18), 218 (30), 217 (25), 204 (19), 203 (41), 202 (47), 59 (100). Anal. calcd for $C_{20}H_{18}Cl_2O_4$, (%): C, 61.08; H, 4.61; Found, %: C, 61.1; H, 4.4.

*1.2.5. Dimethyl 1-chloro-6-fluoro-4-phenyl-3,4-dihydronaphtalene-2,2(1H)-dicarboxylate (**2d**).* Using the procedure described above 248 mg of 4-fluorobenzaldehyde (2 mmol) gave crude product that was purified by column chromatography (petroleum ether : ethyl acetate = 6 : 1) to afford **2d** (328 mg, 87%) as colorless crystals: mp, 122-124 °C. ^{1}H NMR (500 MHz, $CDCl_3$): δ = 2.77 – 2.79 (m, 2H, C(3)H_2), 3.76 (s, 3H, C(10H_3), 3.85 (s, 3H, C(12)H_3), 3.88 – 3.89 (m, 1H, C(4)H), 5.85 (s, 1H, C(1)H), 6.49 (d, *J* = 9.4 Hz, 1H, C(5)H), 6.96 (d, *J* = 8.0 Hz, 1H, C(7)H), 7.22 (d, *J* = 7.5 Hz, 2H, C(14,18)H), 7.28 – 7.32 (m, 1H, C(16)H), 7.36 – 7.43 (m, 3H, C(8,15,17)H). ^{13}C NMR (500 MHz, $CDCl_3$): δ = 31.91 (C(3)), 43.33 (C(4)), 53.24 (C(12)), 53.29 (C(10)), 58.22 (C(1)), 59.56 (C(2)), 114.83 (d, *J* = 22.2 Hz, C(7)), 116.1 (d, *J* = 22.1 Hz, C(5)), 128.81 (C(14,18)), 128.92 (C(15,17)), 131.19 (C(8)), 131.80 (d, *J* = 8.3 Hz, C(8a)), 140.10 (d, *J* = 7.7 Hz, C(4a)), 144.17 (C(13)), 161.59, 163.57 (C(6)), 168.18 (C(11)), 168.62 (C(9)). MS (EI): m/z, % = 341 (5) $[M-Cl]^+$, 340 (17), 281 (81), 280 (67), 249 (30), 222 (35), 221 (37), 220 (34), 203 (12), 202 (20), 196 (14), 173 (7), 146 (19), 145 (19), 125 (14), 59 (100). Anal. calcd for $C_{20}H_{18}ClFO_4$, (%): C, 63.75; H, 4.82; Found, %: C, 64.1; H, 4.8.

*1.2.6. Dimethyl 1-chloro-6-methyl-4-phenyl-3,4-dihydronaphtalene-2,2(1H)-dicarboxylate (**2e**).* Using the procedure described above 240 mg of 4-methylbenzaldehyde (2 mmol) gave crude product that was purified by column chromatography (petroleum ether : ethyl acetate = 6 : 1) to afford **2e** (310 mg, 83%) as colorless crystals: mp, 134-136 ^{o}C. ^{1}H NMR (500 MHz, $CDCl_3$): δ = 2.19 (s, 3H, (C(19)H_3), 2.77 – 2.79 (m, 2H, C(3)H_2), 3.75 (s, 3H, C(10)H_3), 3.85 (s, 3H, C(12)H_3), 3.89 (t, *J* = 9.3 Hz, 1H, C(4)H), 5.86 (s, 1H, C(1)H), 6.61 (s, 1H, C(5)H), 7.07 (d, *J* = 7.8 Hz,1H, C(8)H), 7.24 (d, *J* = 7.4 Hz, 2H, C(14,18)H), 7.28 – 7.38 (m, 4H, C(7,15,16,17)H). ^{13}C NMR (500 MHz, $CDCl_3$): δ = 21.26 (C(19)), 32.52 (C(3)), 43.15 (C(4)), 53.16 (C(12)), 53.19 (C(10)), 58.07 (C(1)), 59.59 (C(2)), 126.83 (C(16)), 128.17 (C(8)), 128.73 (C(14,18)), 128.92 (C(15,17)), 129.82 (C(7)), 130.18 (C(5)), 132.35 (C(6)), 137.23 (C(8a)), 138.79 (C(4a)), 145.21 (C(13)), 168.45 (C(11)), 168.77 (C(9)). MS (EI): m/z, % = 337 (7) $[M-Cl]^+$, 336 (31), 278 (25), 277 (100), 245 (66), 218 (48), 203 (32), 202 (36), 141 (19), 115 (14), 59 (100). Anal. calcd for $C_{21}H_{21}ClO_4$, (%): C, 67.65; H, 5.68; Found, %: C, 67.5; H, 5.8.

*1.2.7. Dimethyl 1,7-dichloro-4-phenyl-3,4-dihydronaphthalene-2,2(1H)-dicarboxylate (**2f**).* Using the procedure described above 281 mg of 3-chlorobenzaldehyde (2 mmol) gave crude product that was purified by column chromatography (petroleum ether : ethyl acetate = 6 : 1) to afford **2f** (330 mg, 84%) as colorless crystals: mp, 149-151 ^{o}C. ^{1}H NMR (500 MHz, $CDCl_3$): δ = 2.71 – 2.81 (m, 2H, C(3)H_2), 3.77 (s, 3H, C(10)H_3), 3.85 (s, 4H, C(4)H, C(12)H_3), 5.77 (s, 1H, C(1)H), 6.75 (d, *J* =8.5 Hz, 1H, C(5)H), 7.12 (dd, *J* = 1.7 Hz, *J* = 8.5 Hz, 1H, C(6)H), 7.20 (d, *J* = 7.3 Hz, 2H, C(14,18)H), 7.28 – 7.31 (m, 1H, C(16)H), 7.35 (t, *J* = 7.1 Hz, 2H, C(15,17)H), 7.41 (s, 1H, C(8)H). ^{13}C NMR (500 MHz, $CDCl_3$): δ = 32.21 (C(3)), 42.81 (C(4)), 53.28 (C(12)), 53.34 (C(10)), 57.89 (C(1)), 59.39 (C(2)), 127.12 (C(16)), 128.79 (C(14,18)), 128.87 (C(15,17)), 129.13 (C(6)), 129.53 (C(8)), 131.33 (C(5)), 132.59 (C(7)), 135.98 (C(4a)), 136.94 (C(8a)), 144.47 (C(13)), 168.06 (C(11)), 168.61 (C(9)). MS (EI): m/z, % = 357 (6)

[M-Cl]$^+$, 356 (27), 324 (7), 297 (100), 296 (76), 265 (40), 238 (23), 218 (45), 217 (29), 203 (46), 202 (57), 101 (11), 59 (100). Anal. calcd for $C_{20}H_{18}Cl_2O_4$, (%): C, 61.08; H, 4.61; Found, %: C, 61.0; H, 4.5.

*1.2.8. Dimethyl 1-chloro-8-fluoro-4-phenyl-3,4-dihydronaphthalene-2,2(1H)-dicarboxylate (**2g**).* Using the procedure described above 248 mg of 2-fluorobenzaldehyde (2 mmol) gave crude product that was purified by column chromatography (petroleum ether : ethyl acetate = 6 : 1) to afford **2g** (305 mg, 81%) as colorless crystals: mp, 144-146 °C. ^{1}H NMR (500 MHz, $CDCl_3$): δ = 2.79 (d, *J* = 9.7 Hz, 2H, C(3)H_2), 3.76 (s, 3H, C(10)H_3), 3.86 (s, 3H, C(12)H_3), 3.91 (t, *J* = 9.7 Hz, 1H, C(4)H), 6.07 (s, 1H, C(1)H), 6.61 (d, *J* = 7.7 Hz, 1H, C(5)H), 6.97 (t, *J* = 8.9 Hz, 1H, C(7)H), 7.13 – 7.17 (q, *J* = 7.7 Hz, 1H, C(6)H), 7.22 (d, *J* = 7.3 Hz, 2H, C(14,18)H), 7.30 (t, *J* = 7.3 Hz, 1H, C(16)H), 7.36 (t, *J* = 7.3 Hz, 2H, C(15,17)H). ^{13}C NMR (500 MHz, $CDCl_3$): δ = 32.12 (C(3)), 43.14 (C(4)), 51.80 (d, *J* = 5.7 Hz, C(1)), 53.27 (C(12)), 53.31 (C(10)), 59.06 (C(2)), 113.38 (d, *J* = 20.9 Hz, C(7)), 123.83 (d, *J* = 14.7 Hz, C(8a)), 125.35 (d, *J* = 3.3 Hz, C(5)), 127.09 (C(16)), 128.82 (C(14,18)), 128.84 (C(15,17)), 129.78 (d, *J* = 9.1 Hz, C(6)), 139.75 (C(4a)), 144.50 (C(13)), 159.23, 161.22 (C(8)), 168.07 (C(11)), 168.55 (C(9)). MS (EI): m/z, % = 341 (4) [M-Cl]$^+$, 340 (18), 281 (100), 280 (80), 249 (20), 237 (13), 222 (38), 221 (31), 202 (23), 146 (11), 59 (25). Anal. calcd for $C_{20}H_{18}ClFO_4$, (%): C, 63.75; H, 4.82; Found, %: C, 63.3; H, 4.9.

*1.2.9. Dimethyl 2-(2-chloro-2-phenylethyl)malonate (**3**)* [21]. Using the procedure described but in the absence of benzaldehyde derivative gave crude product that was purified by column chromatography (petroleum ether : ethyl acetate = 6 : 1) to afford **4** (305 mg, 81%) as a colourless oil. ^{1}H NMR (500 MHz, $CDCl_3$): δ = 2.66 (t, *J* = 7.3 Hz, 2H, C(2)H_2), 3.69 (t, *J* = 7.3 Hz, 1H, C(3)H), 3.77 (s, 3H, C(7)H_3), 3.78 (s, 3H, C(5)H_3), 4.97 (t, *J* = 7.3 Hz, 1H, C(1)H), 7.33 – 7.42 (m, 5H, C(9,10,11,12,13)H). ^{13}C NMR (500 MHz, $CDCl_3$): δ = 38.78 (C(2)), 49.41 (C(3)), 52.79 (C(5)), 52.81 (C(7)),

60.85 (C(1)), 126.92 (C(9,13)), 128.70 (C(11)), 128.80 (C(10,12)), 140.45 (C(8)), 168.99 (C(4)), 169.12 (C(6)). MS (EI): m/z, % = 271 (<1) $[M]^+$, 270 (2), 171 (3), 132 (100), 115 (61), 100 (25), 59 (31).

Acknowledgements

This work was financially supported by the Russian Science Foundation (grant No. 22-73-00251).

References

1. Fringuelli, F.; Germani, R.; Pizzo, F.; Savelli, G. One-pot two-steps synthesis of 1, 2-diol. *Synthetic Communications* **1989**, *19*, 1939–1943.
2. Doppiu, A.; Salzer, A. A New Route to Cationic Half-Sandwich Ruthenium (II) Complexes with Chiral Cyclopentadienylphosphane Ligands. *European Journal of Inorganic Chemistry* **2004**, *2004*, 2244–2252.
3. Sapeta, K.; Kerr, M.A. The cycloaddition of nitrones with homochiral cyclopropanes. *The Journal of Organic Chemistry* **2007**, *72*, 8597–8599.
4. Frisch, M.J. et al. Gaussian 09, Revision A.1. Gaussian, Inc., Wallingford, CT, 2009.
5. Sheldrick, G.M. SHELXT-Integrated Space-Group and Crystal-Structure Determination. *Acta Crystallographica Section A* **2015**, *A71*, 3-8.
6. Sheldrick, G.M. Crystal structure refinement with SHELXL. *Acta Crystallographica Section C* **2015**, *C71*, 3-8.
7. Dolomanov, O.V.; Bourhis, L.J.; Gildea, R.J.; Howard, J.A.K. and Puschmann, H. OLEX2: A Complete Structure Solution, Refinement and Analysis Program. *Journal of Applied Crystallography* **2009**, *42*, 339-341.

^{13}C-NMR spectrum of dimethyl 1-chloro-6-iodo-4-phenyl-3,4-dihydronaphtalene-2,2(1*H*)-dicarboxylate (3a)

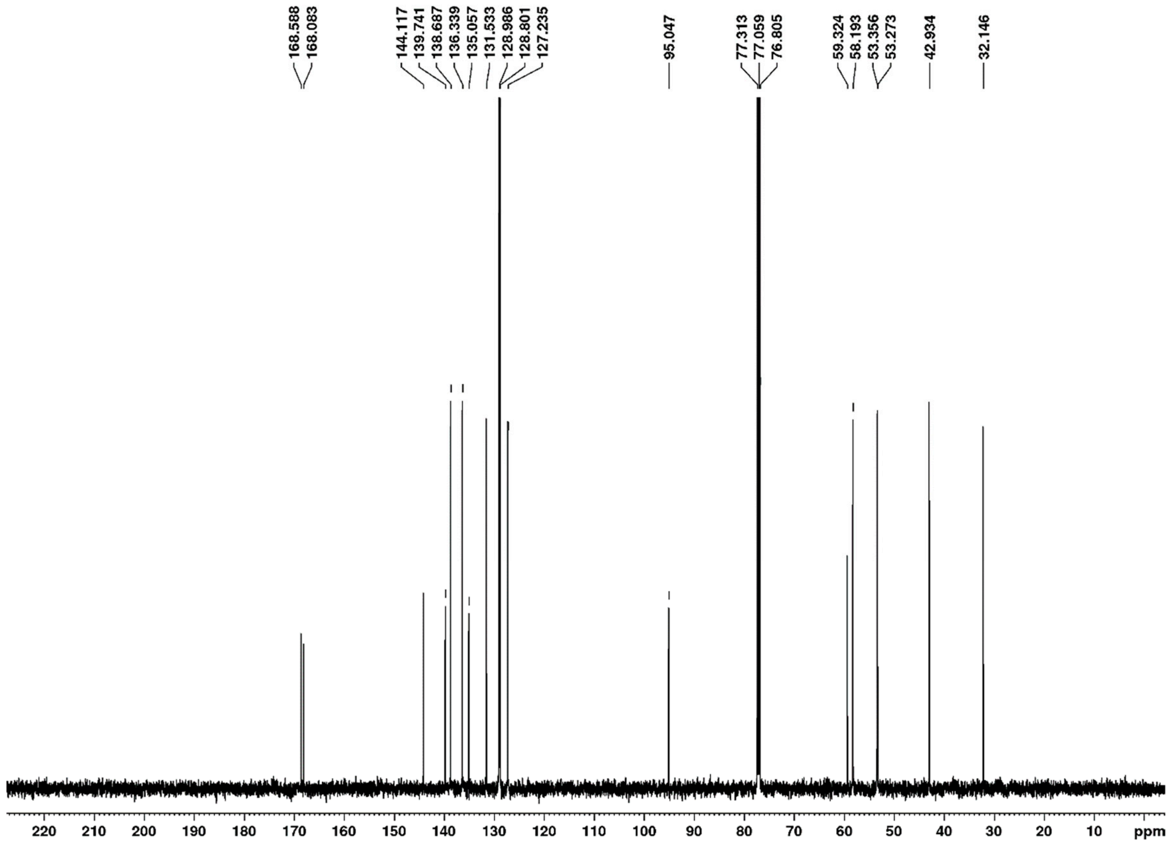

^{1}H-NMR spectrum of dimethyl 1-chloro-6-iodo-4-phenyl-3,4-dihydronaphtalene-2,2(1*H*)-dicarboxylate (3a)

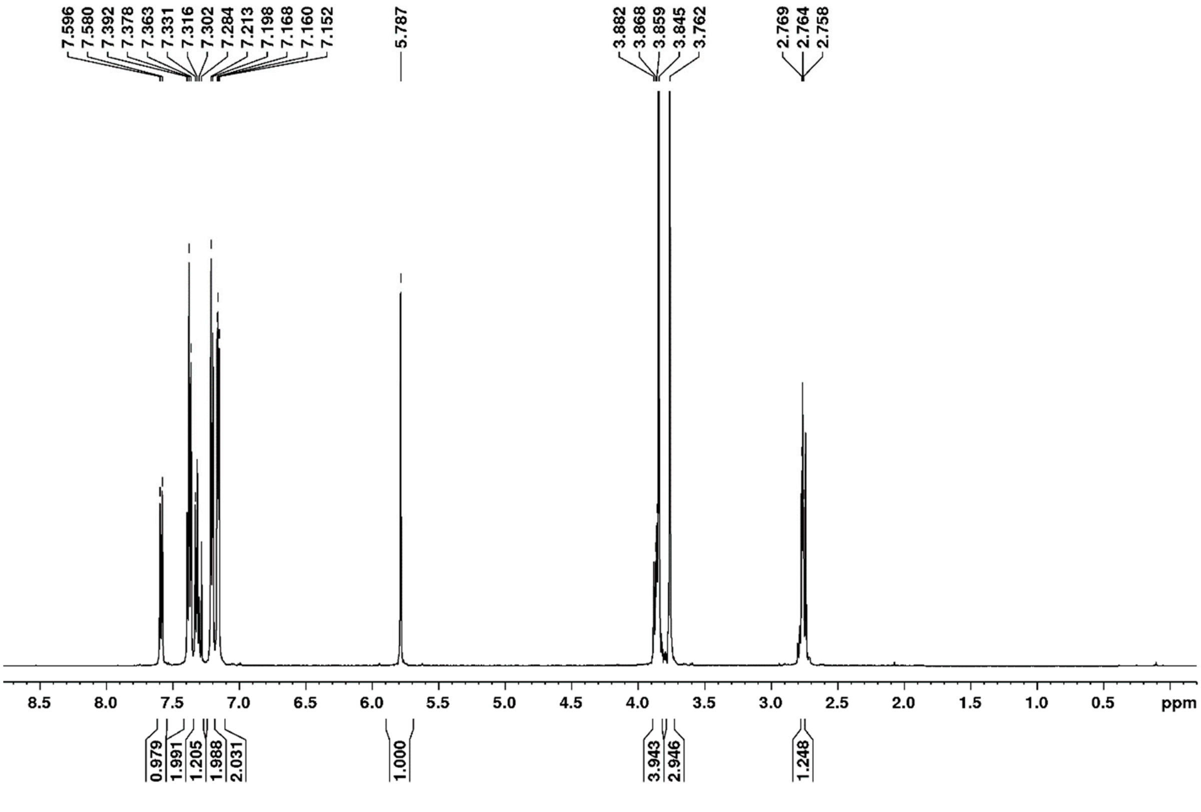

^{13}C-NMR spectrum of dimethyl 6-bromo-1-chloro-4-phenyl-3,4-dihydronaphtalene-2,2(1*H*)-dicarboxylate (3b)

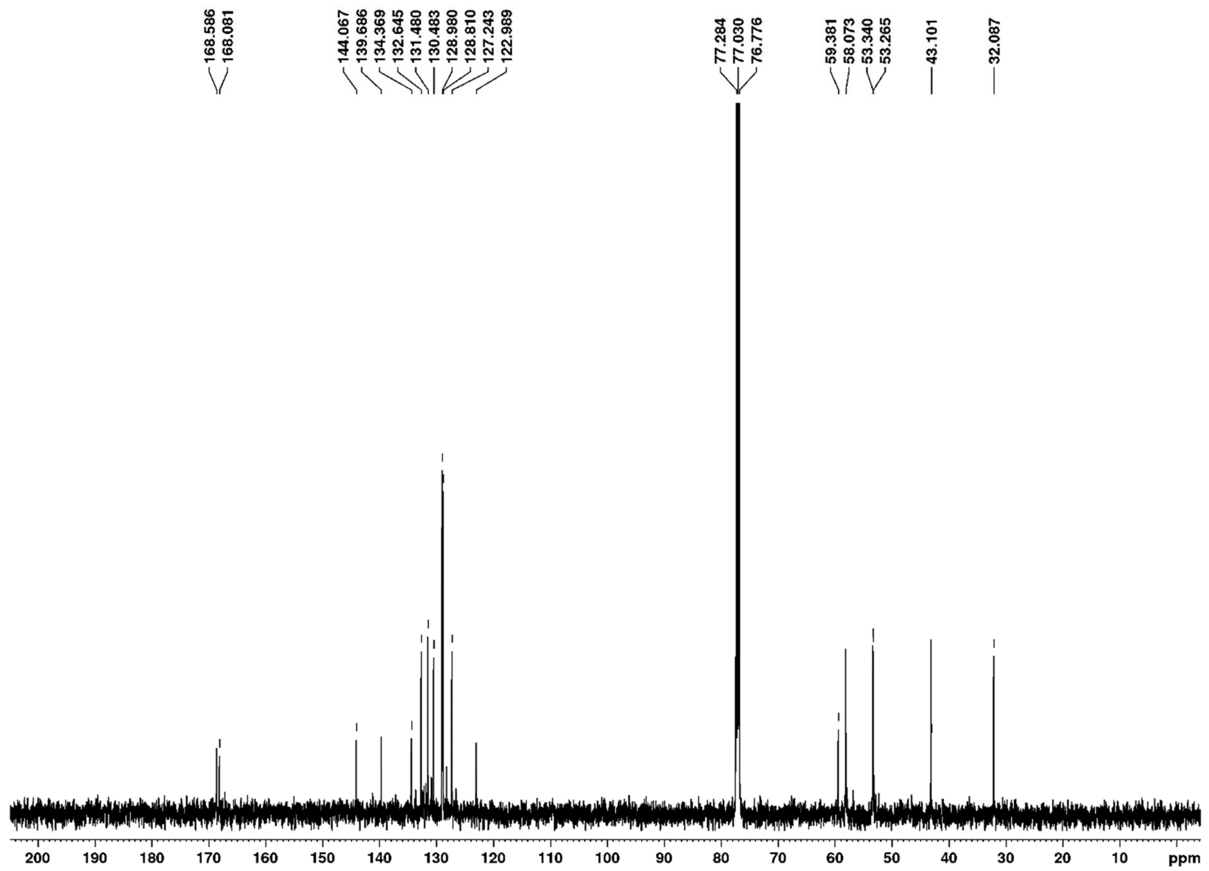

^{1}H-NMR spectrum of dimethyl 6-bromo-1-chloro-4-phenyl-3,4-dihydronaphtalene-2,2(1*H*)-dicarboxylate (3b)

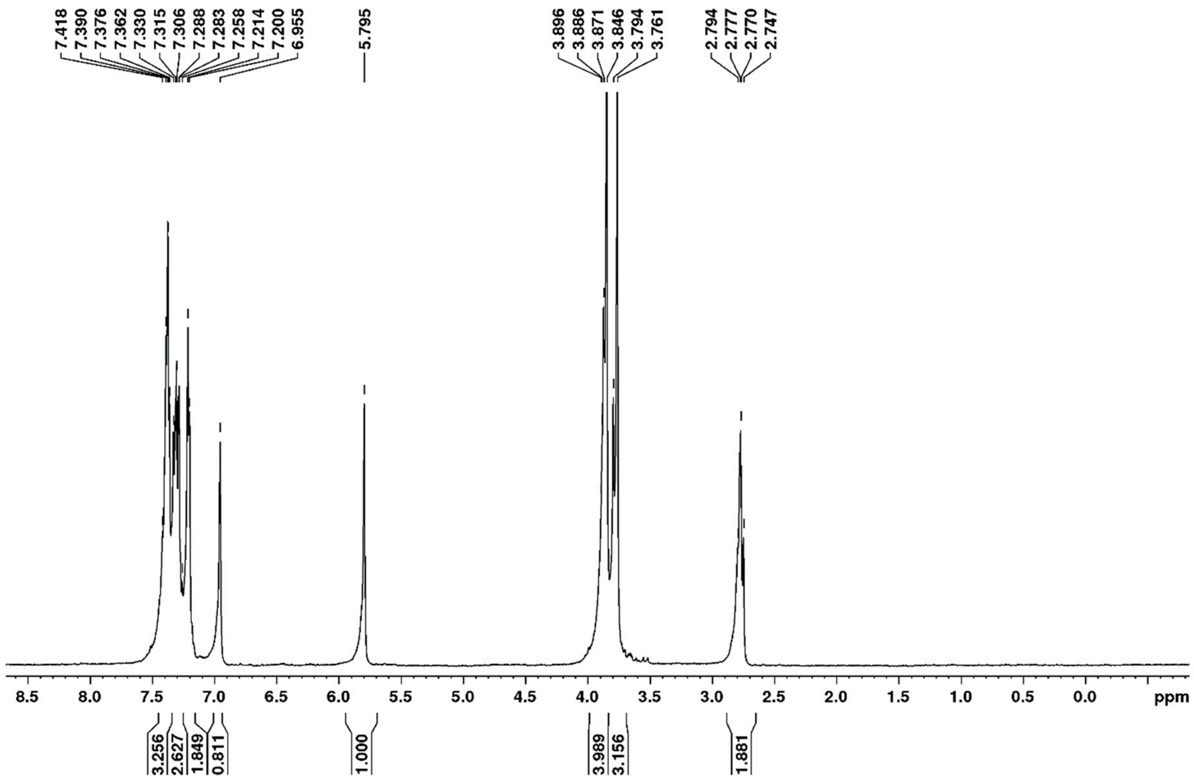

^{13}C-NMR spectrum of dimethyl 1,6-dichloro-4-phenyl-3,4-dihydronaphtalene-2,2(1*H*)-dicarboxylate (3c)

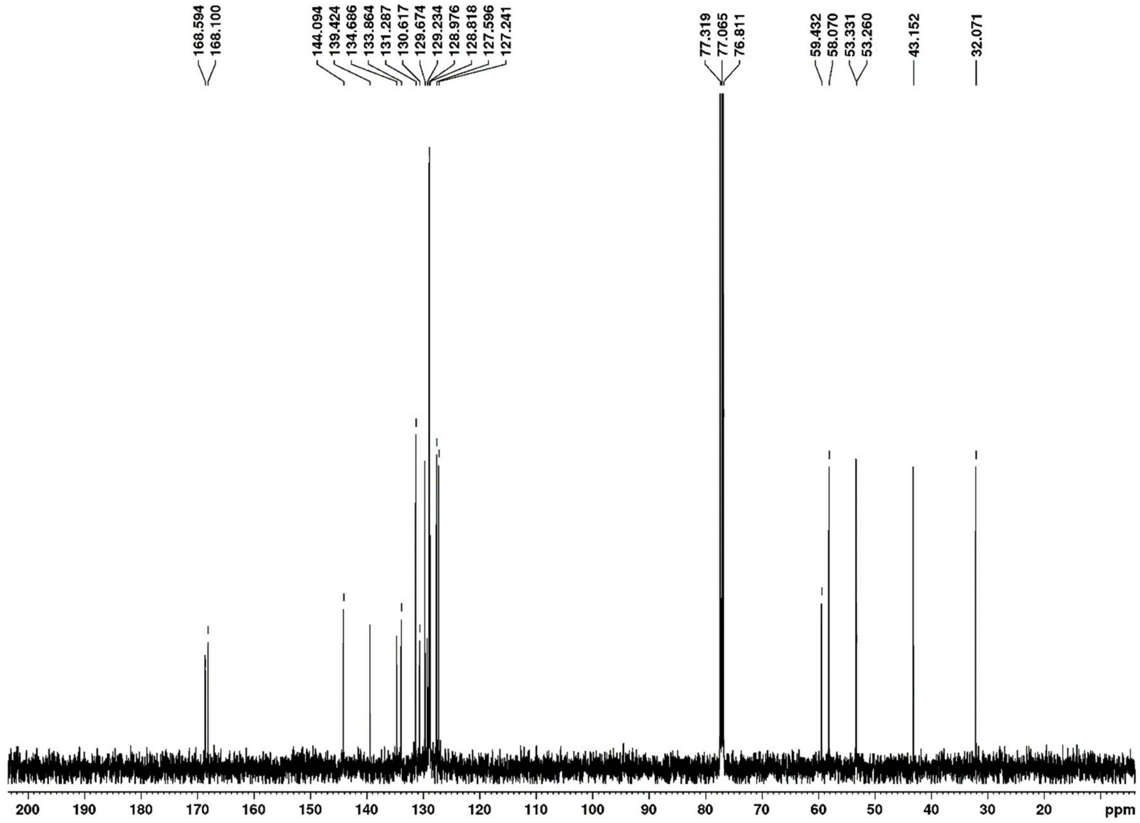

^{1}H-NMR spectrum of dimethyl 1,6-dichloro-4-phenyl-3,4-dihydronaphtalene-2,2(1H)-dicarboxylate (3c)

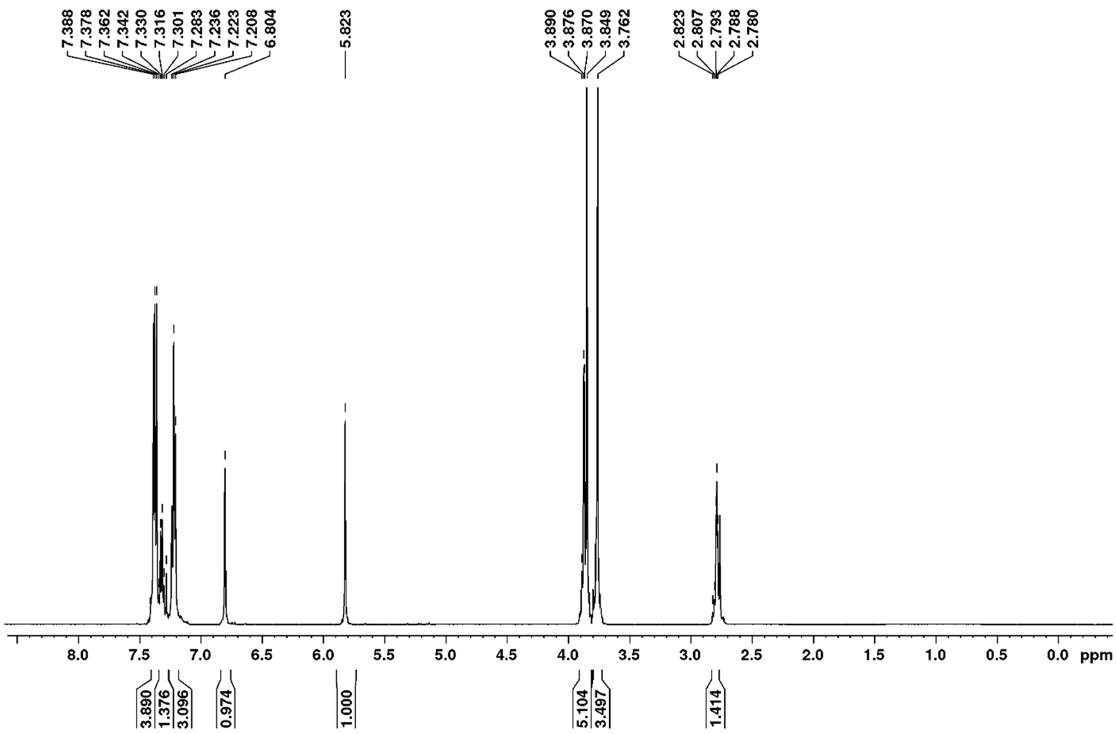

^{13}C-NMR spectrum of dimethyl 1-chloro-6-fluoro-4-phenyl-3,4-dihydronaphtalene-2,2(1*H*)-dicarboxylate (3d)

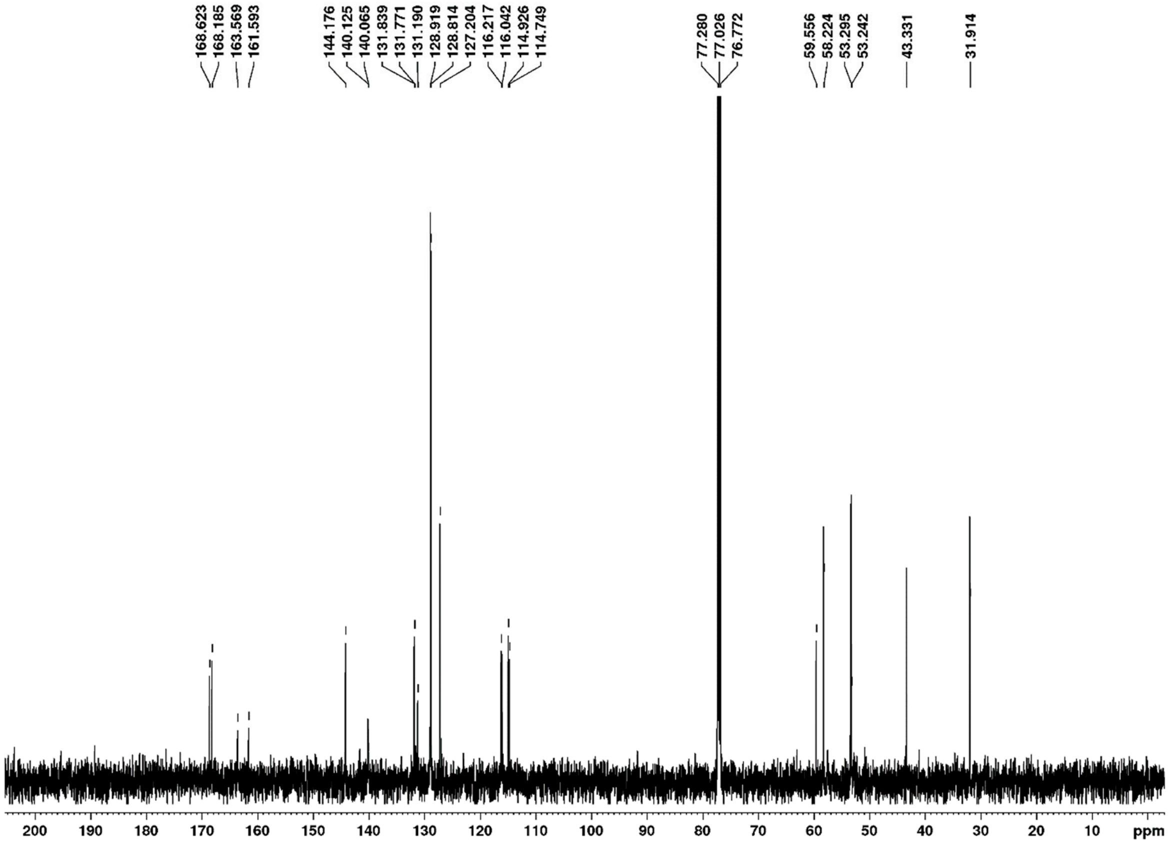

^{1}H-NMR spectrum of dimethyl 1-chloro-6-fluoro-4-phenyl-3,4-dihydronaphtalene-2,2(1*H*)-dicarboxylate (3d)

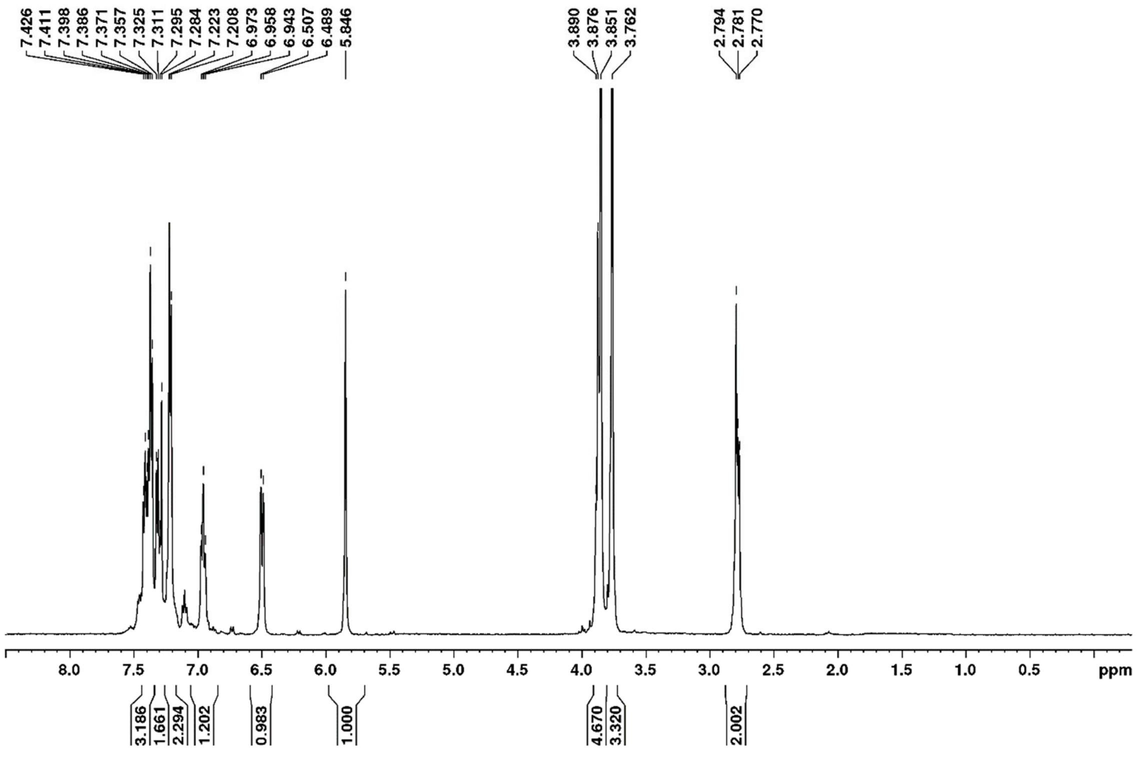

^{13}C-NMR spectrum of dimethyl 1-chloro-6-methyl-4-phenyl-3,4-dihydronaphtalene-2,2(1*H*)-dicarboxylate (3e)

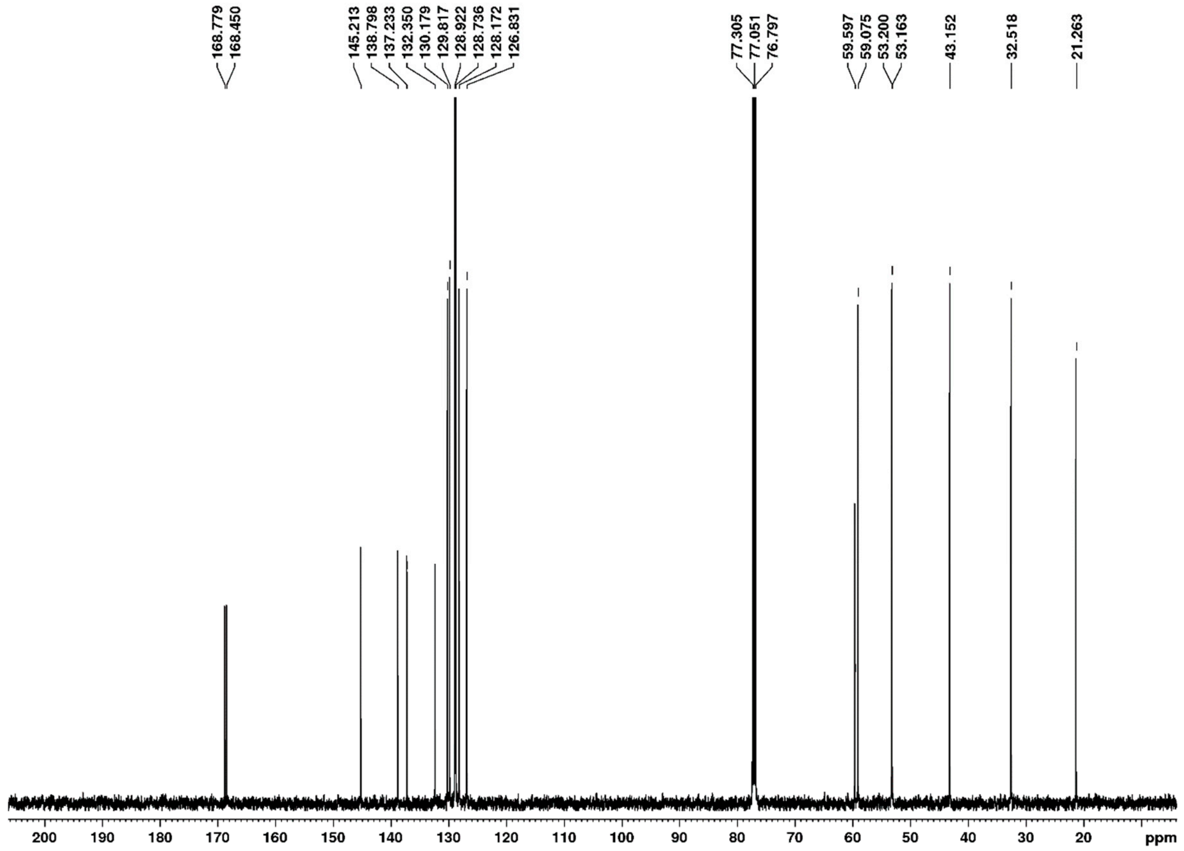

^{1}H-NMR spectrum of dimethyl 1-chloro-6-methyl-4-phenyl-3,4-dihydronaphtalene-2,2(1*H*)-dicarboxylate (3e)

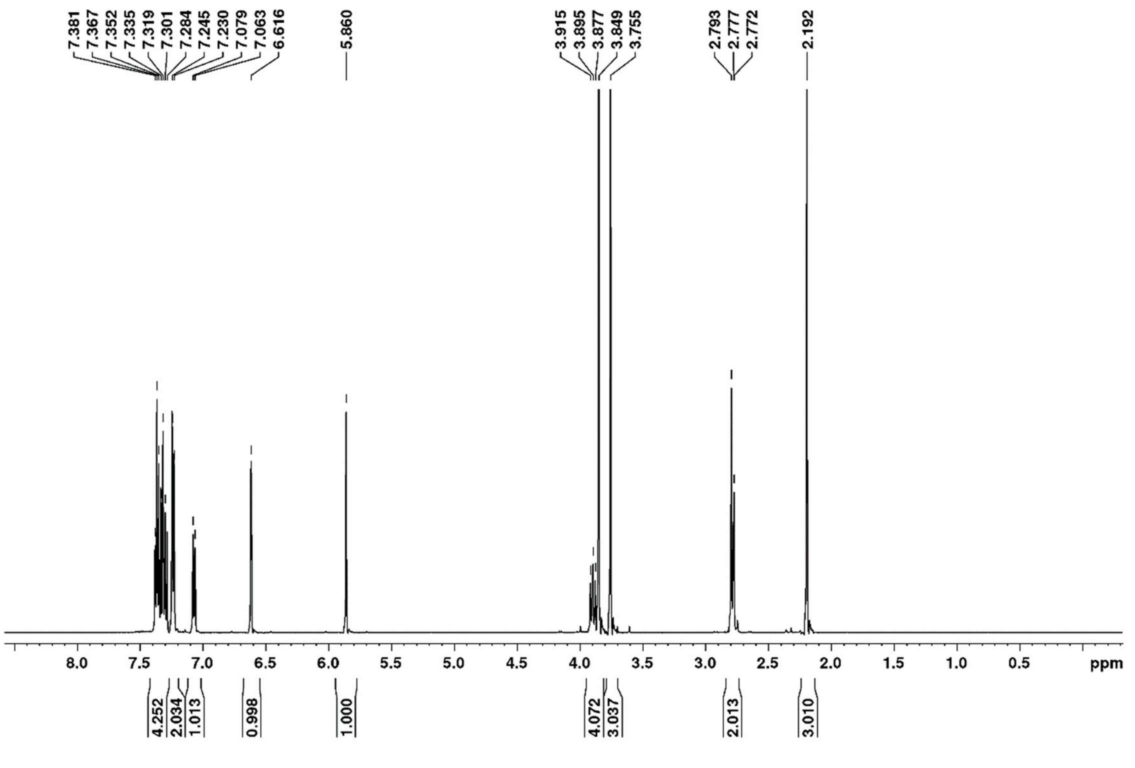

^{13}C-NMR spectrum of dimethyl 1,7-dichloro-4-phenyl-3,4-dihydronaphthalene-2,2(1*H*)-dicarboxylate (3f)

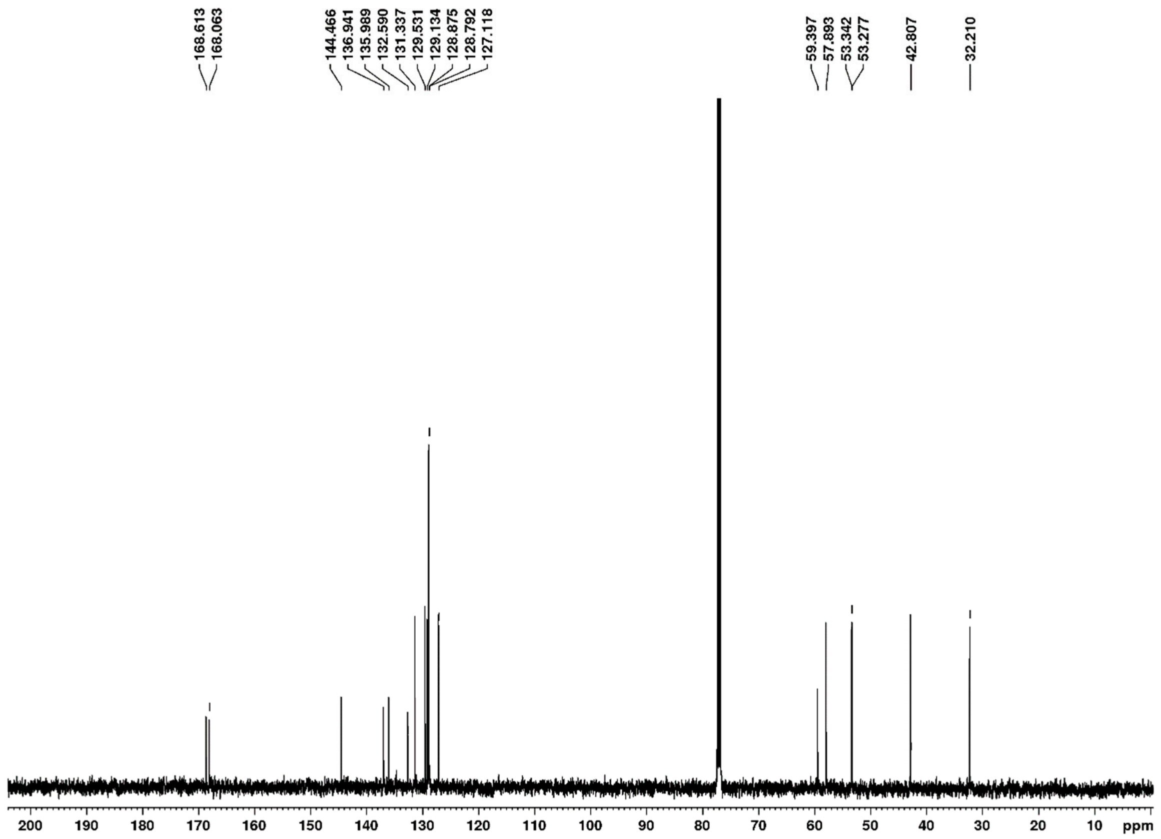

^{1}H-NMR spectrum of dimethyl 1,7-dichloro-4-phenyl-3,4-dihydronaphthalene-2,2(1*H*)-dicarboxylate (3f)

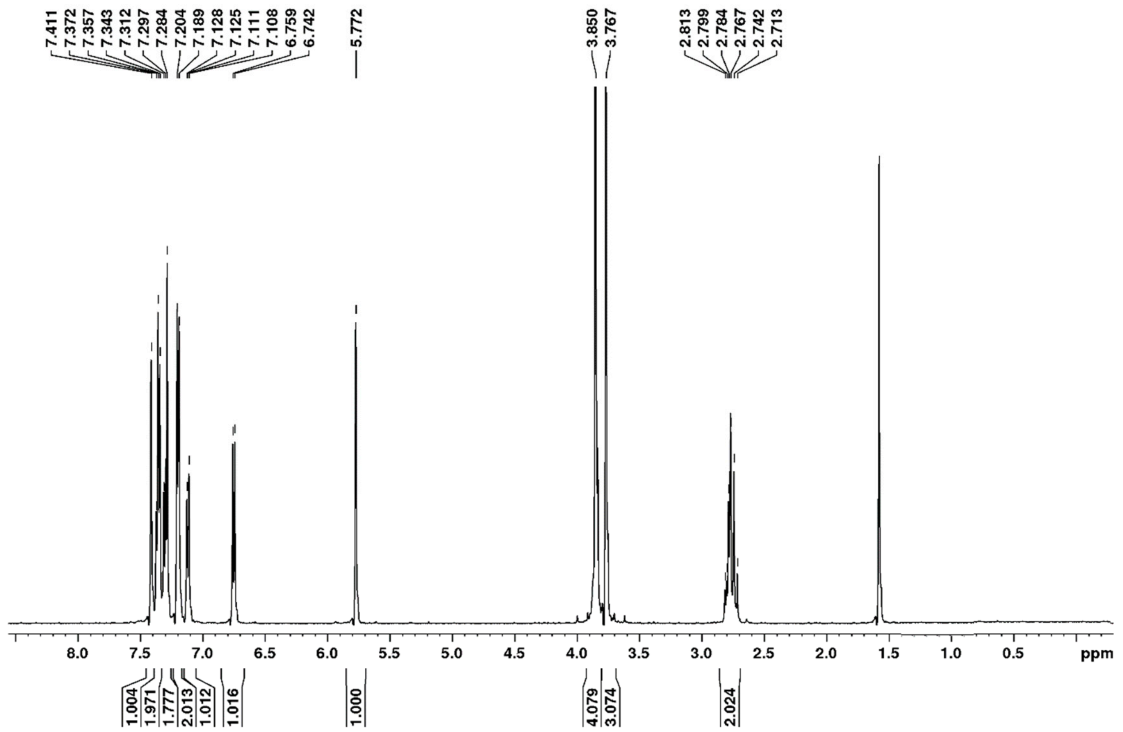

^{13}C-NMR spectrum of 1-chloro-8-fluoro-4-phenyl-3,4-dihydronaphthalene-2,2(1*H*)-dicarboxylate (3g)

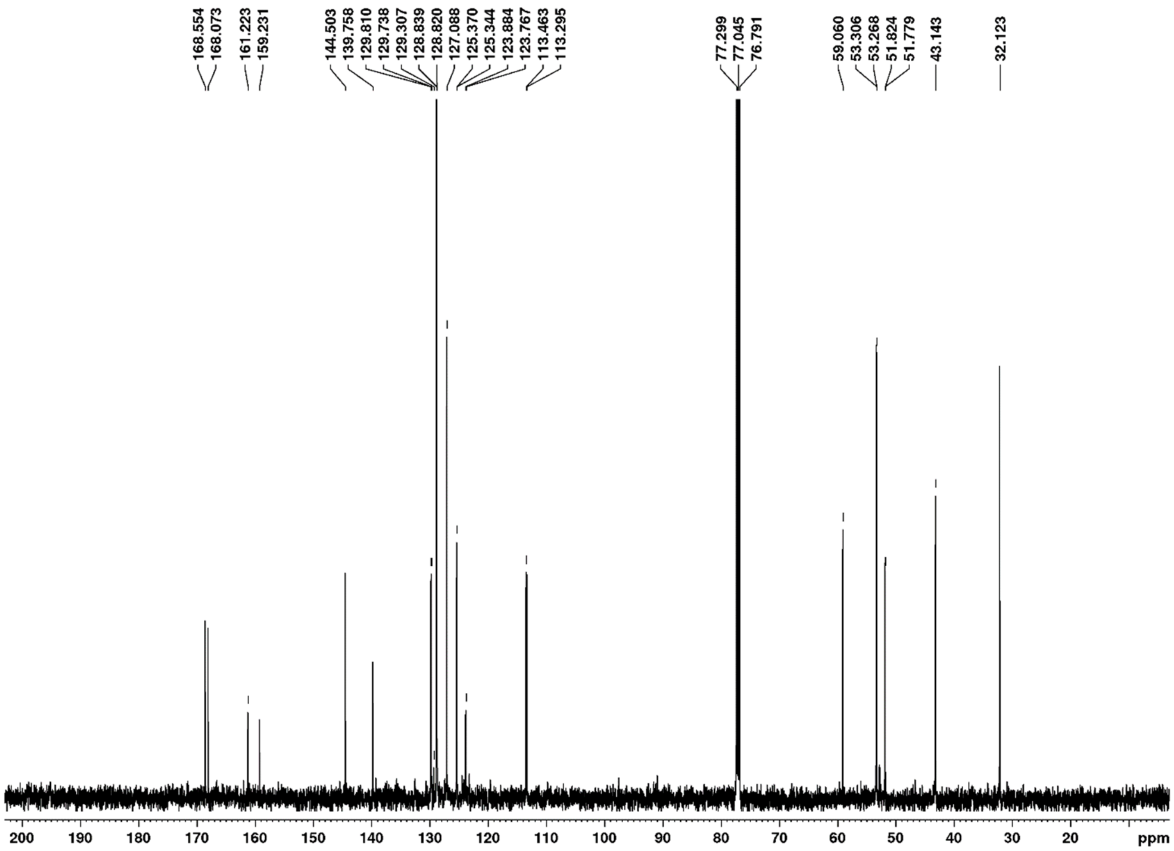

^{1}H-NMR spectrum of 1-chloro-8-fluoro-4-phenyl-3,4-dihydronaphthalene-2,2(1*H*)-dicarboxylate (3g)

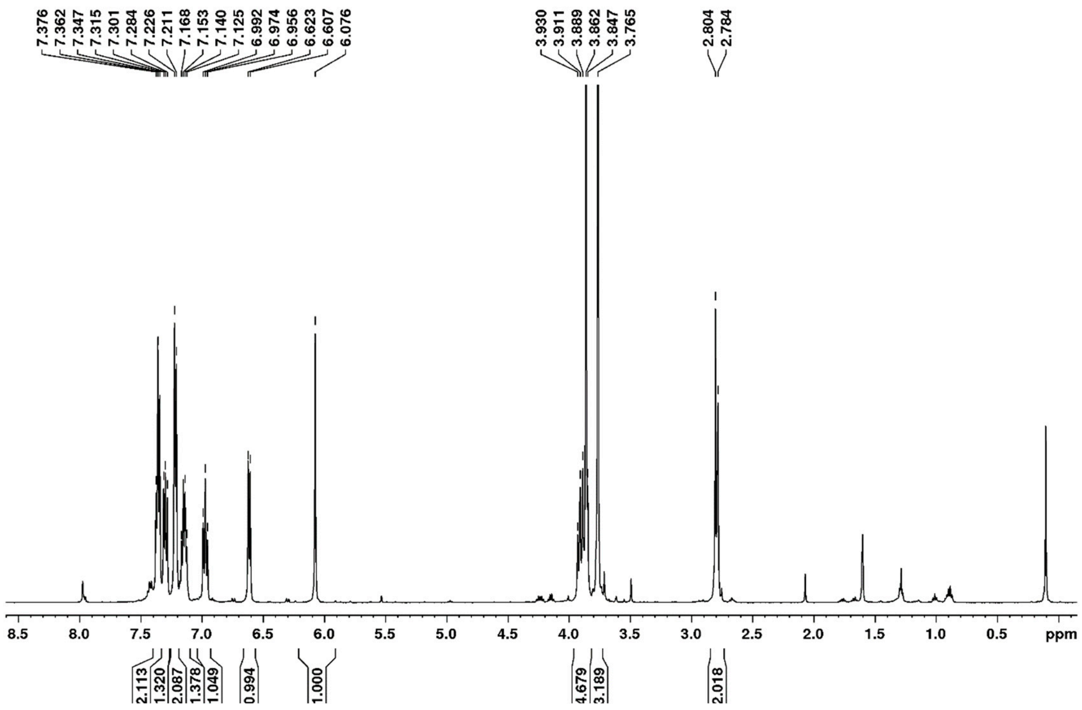

^{13}C-NMR spectrum of dimethyl 2-(2-chloro-2-phenylethyl)malonate (4)

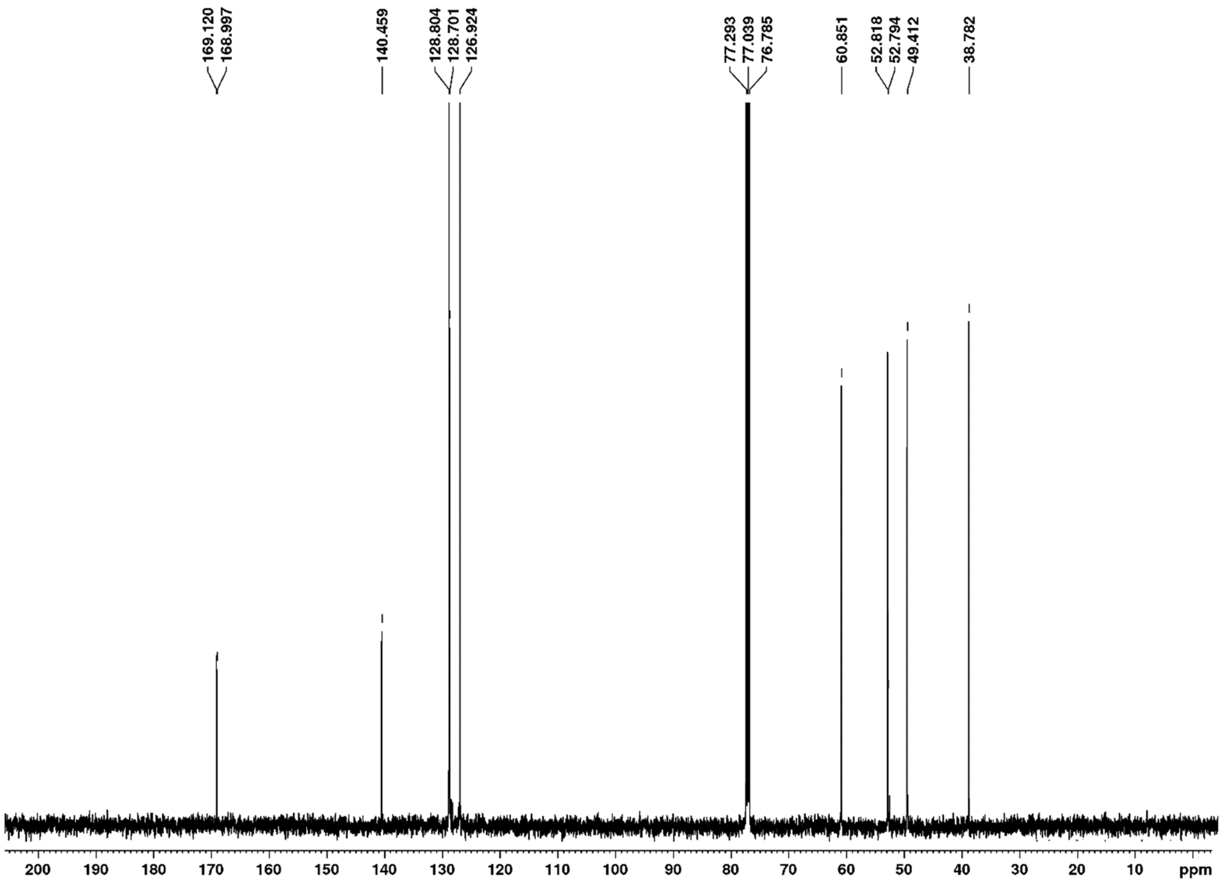

^{1}H-NMR spectrum of dimethyl 2-(2-chloro-2-phenylethyl)malonate (3g)

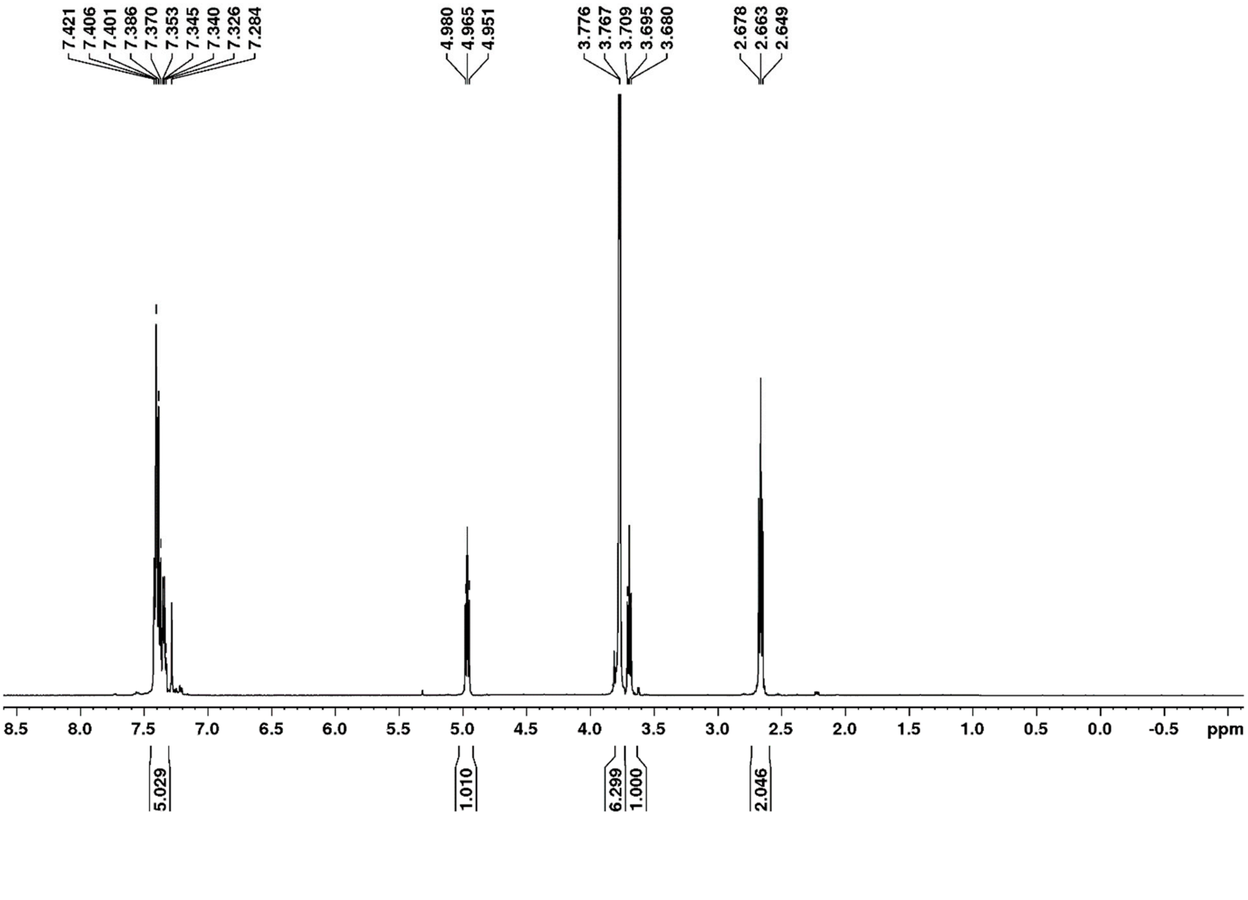

Supplement: Supplementary file 1 [file molecules-29-02715-s001.zip › molecules-3033901-supplementary.pdf]
